# The postprandial secretion of peptide YY_1‐36_ and _3‐36_ in obesity is differentially increased after gastric bypass versus sleeve gastrectomy

**DOI:** 10.1111/cen.14846

**Published:** 2022-11-28

**Authors:** Anna M. Kowalka, Kleopatra Alexiadou, Joyceline Cuenco, Rosemary E. Clarke, James Minnion, Emma L. Williams, Paul Bech, Sanjay Purkayastha, Ahmed R. Ahmed, Zoltan Takats, Harry J. Whitwell, Maria Gomez Romero, Stephen R. Bloom, Stephane Camuzeaux, Matthew R. Lewis, Bernard Khoo, Tricia M.‐M. Tan

**Affiliations:** ^1^ Section of Diabetes, Endocrinology and Metabolism, Department of Metabolism, Digestion and Reproduction Imperial College London London UK; ^2^ Blood Sciences Department Raigmore Hospital Inverness UK; ^3^ Department of Clinical Biochemistry, North West London Pathology Charing Cross Hospital London UK; ^4^ Department of Surgery and Cancer Imperial College Healthcare NHS Trust London UK; ^5^ Section of Bioanalytical Chemistry, Department of Metabolism, Digestion and Reproduction Imperial College London London UK; ^6^ National Phenome Centre Imperial College London London UK; ^7^ Endocrinology, Division of Medicine University College London London UK

**Keywords:** PYY, gastric bypass, type 2 diabetes, obesity, LC‐MS/MS

## Abstract

**Objectives:**

Peptide tyrosine tyrosine (PYY) exists as two species, PYY_1‐36_ and PYY_3‐36_, with distinct effects on insulin secretion and appetite regulation. The detailed effects of bariatric surgery on PYY_1‐36_ and PYY_3‐36_ secretion are not known as previous studies have used nonspecific immunoassays to measure total PYY. Our objective was to characterize the effect of sleeve gastrectomy (SG) and Roux‐en‐Y gastric bypass (RYGB) on fasting and postprandial PYY_1‐36_ and PYY_3‐36_ secretion using a newly developed liquid chromatography‐tandem mass spectrometry (LC‐MS/MS) assay.

**Design and Subjects:**

Observational study in 10 healthy nonobese volunteers and 30 participants with obesity who underwent RYGB (*n* = 24) or SG (*n* = 6) at the Imperial Weight Centre [NCT01945840]. Participants were studied using a standardized mixed meal test (MMT) before and 1 year after surgery. The outcome measures were PYY_1‐36_ and PYY_3‐36_ concentrations.

**Results:**

Presurgery, the fasting and postprandial levels of PYY_1‐36_ and PYY_3‐36_ were low, with minimal responses to the MMT, and these did not differ from healthy nonobese volunteers. The postprandial secretion of both PYY_1‐36_ and PYY_3‐36_ at 1 year was amplified after RYGB, but not SG, with the response being significantly higher in RYGB compared with SG.

**Conclusions:**

There appears to be no difference in PYY secretion between nonobese and obese volunteers at baseline. At 1 year after surgery, RYGB, but not SG, is associated with increased postprandial secretion of PYY_1‐36_ and PYY_3‐36_, which may account for long‐term differences in efficacy and adverse effects between the two types of surgery.

## 1 INTRODUCTION

Peptide tyrosine tyrosine (PYY) is a member of the PP‐fold family which includes neuropeptide Y (NPY) and pancreatic polypeptide (PP). PYY exists in two active species, PYY_1‐36_ and PYY_3‐36_, with dipeptidyl peptidase IV (DPP‐4) converting the former to the latter by removal of the N‐terminal dipeptide.^1^ The C‐terminal amidation of both species is essential for bioactivity. PYY_1‐36_ binds to all G‐protein‐coupled neuropeptide Y receptor subtypes in humans (Y1, Y2, Y4, Y5), whereas PYY_3‐36_ shows selective and high affinity for subtype Y2.^2^

These species of PYY have distinct effects on physiology. PYY_1‐36_ treatment and Y1 receptor activation stimulates insulin secretion and suppresses glucagon secretion.^3^ PYY_3‐36_ does not have any effects on acute insulin secretion in response to an IV glucose stimulus,^4^ consistent with the absence of Y2 in islets.^5^ Moreover, intraislet/paracrine PYY_1‐36_, secreted by a subpopulation of cells in the periphery of the islet, maintains the health of beta‐cells,^6^ via antiapoptotic/pro‐proliferative actions.^2^ PYY_3‐36_ is not involved as it is unable to rescue the negative effects of deleting intra‐islet PYY secretion.^6^ Instead, PYY_3‐36_ regulates appetite by providing feedback inhibition after eating^7^, ^8^ whereas PYY_1‐36_ does not inhibit food intake.^9^ Specific blockade of Y2 receptors inhibits the anorectic effect of PYY_3‐36_.^10^

Postprandial circulating levels of total PYY rise markedly after Roux‐en‐Y gastric bypass (RYGB) surgery.^11^ This elevation in postprandial PYY secretion, in synergetic combination with the anorectic gut hormones GLP‐1 and oxyntomodulin (OXM), is conjectured to suppress appetite and food intake after eating, thus leading to weight loss.^12^ To date, most studies examining the levels of PYY after bariatric surgery have used total PYY immunoassays which do not generally distinguish between PYY_1‐36_ and PYY_3‐36_ and which could be biased by interference from nonspecific binding to related PP‐fold peptides or nonactive PYY fragments. Liquid chromatography‐tandem mass spectrometry (LC‐MS/MS) has a specificity advantage over immunoassays and can distinguish between peptide hormones that differ by only a single amino acid, allowing for multiplexed quantification of highly similar peptide species.^13^ To date, described quantitative LC‐MS/MS‐based assays for PYY include an assay that detects only one species of PYY (PYY_1‐36_) using trypsin to generate the detected fragment PYY_1‐19_. This assay was reported to have an analytical sensitivity of 5 ng/ml (1.15 nmol/L) which is insufficient to detect the pmol/L concentrations of PYY in plasma.^14^ A more recent publication by Reverter‐Branchat et al. described an LC‐MS/MS assay detecting all PP‐fold peptides including PYY_1‐36_ and PYY_3‐36_. This assay relies on immunoaffinity capture and a microflow LC to increase analytical sensitivity to 1.5 pmol/L. However, the immunocapture approach can increase the work required per assay and markedly reduces peptide recovery.^15^ The aim of our study was to devise a specific, sensitive and quantitative multiplexed LC‐MS/MS assay for PYY_1‐36_ and PYY_3‐36_, and to use this to analyse the changes in the secretion of these hormones before and after RYGB and sleeve gastrectomy (SG).

## 2 MATERIALS AND METHODS

### 2.1 Study subjects

The study was carried out at the National Institute for Health Research Imperial Clinical Research Facility (ICRF) at Hammersmith Hospital. This study is part of a series of experimental medicine studies on the mechanisms of bariatric surgery (ClinicalTrials.gov NCT01945840) which has been described elsewhere.^12^, ^16^ The healthy volunteer samples were derived from the control ‘no treatment’ arm of a previous study.^17^ Ethical approval was obtained from the UK National Health Service (NHS) Health Research Authority West London National Research Ethics Committee (references 13/LO/1510, 17/LO/0126 and 17/LO/1323). For the study in people undergoing bariatric surgery, subjects with obesity with BMI > 35 kg/m^2^ who were eligible for bariatric surgery under the criteria set out by the UK National Institute for Health and Care Excellence^18^ were recruited through the Imperial Weight Centre (IWC, a tertiary centre for obesity management) for a longitudinal, prospective observational study. If diabetic, patients were taking either no treatment or a single oral hypoglycaemic agent and were asked to discontinue the treatment 2 weeks before the enrolment in the study. All participants provided written informed consent, and the study was conducted according to the principles of the Declaration of Helsinki.

Participants attended the research unit for a baseline visit before the operation and were subsequently studied at 1 year. For each visit, the volunteers attended the ICRF at 08:00 AM after a 10‐h overnight fast. After anthropometric measurements, a cannula was inserted in the antecubital fossa and blood samples were collected at a fasting state (baseline) and at 15, 30, 60, 120 and 180 min after a standardized mixed meal test (MMT—Ensure Plus; Abbott Nutrition). The MMT was consumed over 10 min (a serving volume of 220 ml containing 330 kcal from 13.8 g of protein, 10.8 g of fat and 44.4 g of carbohydrates).

### 2.2 Assays for glucose, insulin, HbA1c

Samples for glucose and insulin analysis were collected in sodium fluoride and clotting activator tubes, respectively, and analysed at the North West London Pathology laboratory on Alinity analysers (Abbott) with coefficient of variation (CV) of <5% and <10%, respectively. Whole blood was collected in K_3_EDTA tubes for glycated haemoglobin (HbA1c) analysis on a G8 HPLC analyser (Tosoh Bioscience N.V.) with CV of <2%.

### 2.3 LC‐MS/MS assay for PYY_1‐36_ and _3‐36_

Plasma samples for gut hormones were collected in lithium heparin tubes containing Aprotinin (Nordic Pharma) and the DPP‐4 inhibitor Diprotin A (Enzo Life Sciences). Samples were placed on ice and centrifuged at 4°C within 10 min of collection. They were stored at −80°C until being thawed once for analysis.

All reagents used were LC‐MS grade and included: ultrapure water (Optima® LC/MS grade, Fluka/Optima), methanol (MeOH), acetonitrile (ACN) and propan‐2‐ol (IPA) from LC‐MS Chromasolv (Honeywell Research Chemicals), ammonium hydroxide solution, 28.0%–30.0% (NH_4_OH, Honeywell/Fluka), acetic acid ReagentPlus® (HAc, Sigma‐Aldrich), formic acid for mass spectrometry (FA, Honeywell/Fluka) and bovine serum albumin (BSA) (Sigma‐Aldrich). PYY_1‐36_ and PYY_3‐36_ were purchased from Bachem (Switzerland) for use as calibration components whereas synthetic peptide control samples were obtained from Phoenix Pharmaceuticals, Inc. Isotopically labelled internal standards for PYY_1‐36_: YPIKPEAPGEDASPEE‐[U‐^13^C_6_,^15^N‐Leu]‐NRYYAS‐[U‐^13^C_6_,^15^N‐Leu]‐RHY‐[U‐^13^C_6_,^15^N‐Leu]‐N‐[U‐^13^C_6_,^15^N‐Leu]‐VTRQRY‐amide with molecular weight (MW) of 4335.3 and PYY_3‐36_: IKPEAPGEDASPEE‐[U‐^13^C_6_,^15^N‐Leu]‐NRYYAS‐[U‐^13^C_6_,^15^N‐Leu]‐RHY‐[U‐^13^C_6_,^15^N‐Leu]‐N‐[U‐^13^C_6_,^15^N‐Leu]‐VTRQRY‐amide with MW of 4075.1 were custom made by Cambridge Research Biochemicals. Deamidated (without C‐terminal amide group) PYY_1‐36_ and PYY_3‐36_, PYY_1‐34_, PYY_3‐34_ were obtained from WuXi AppTec (China). NPY and PP were purchased from Bachem (Switzerland).

To create the calibration mixture, both peptides were first weighed and dissolved in a 1:4 ratio (volume/volume) of acetonitrile and water with 0.1% formic acid in Clear‐view™ Snap‐Cap microtubes (Sigma‐Aldrich) to give 1 mg/ml stock solutions. These were further diluted with 20 µg/ml BSA prepared in a 1:2:7 ratio of methanol, acetic acid and water (20BMA) with a final concentration of 1 µmol/L for each peptide. The combined peptide mixture with 1 µmol/L PYY_1‐36_ and 1  µmol/L of PYY_3‐36_ was prepared by further dilution with 20BMA to 2.5  nmol/L and used to generate the following calibration curve: 2, 5, 10, 25, 50 and 100 pmol/L for both PYY species. Phoenix‐sourced quality control (QC) materials were prepared in the same way to give sample concentrations at: 12, 40 and 80 pmol/L for QC Low, QC Medium and QC High, respectively. The calibration standards and QC samples were stored at −80°C until the day of analysis.

### 2.4 Sample extraction procedure

A total of 10 μl of combined internal standards and 720 µl of 75% ACN, 0.1% NH_4_OH were added to 250 μl of calibrator/control/patient sample. Following 15 s vortexing, the samples were then centrifuged at 4°C at 6000*g* for 10 min and 800 µl of supernatant were transferred and evaporated to dryness at 40°C. Extracts were reconstituted with 500 μl of 5% NH_4_OH and transferred into corresponding wells of preconditioned Oasis® MAX μElution Plate (Waters). The samples were pulled through at low vacuum using Waters 96‐well Extraction Plate Vacuum Manifold and the wells were washed with 200 μl of 5% NH_4_OH followed by 200 μl of 60% ACN. The elution step was carried out by two‐step addition of 25 μl aliquots of 30% ACN, 1% FA into each well and collecting eluent into a plate containing 50 µl of 20BMA. The plate was sealed, and the content of wells mixed well.

### 2.5 Instrument settings

The extracts were analysed using a Xevo‐TQS (Waters Corp.) triple quadruple mass spectrometer and Acquity UPLC system controlled by MassLynx® V4.1 software (Waters Corp.). For each sample, 20 µl were injected onto a 130 Å Waters ACQUITY UPLC Peptide BEH C18 1.7 µm (2.1 × 100 mm) column thermo‐controlled at 40°C. The mobile phase system consisted of 0.1% FA in water (A) and 0.1% FA in ACN (B). Initial chromatographic conditions were set to 80/20 (A/B) at a flow rate of 0.15 ml/min. A linear gradient elution was conducted over 8 min, reaching 70/30 (A/B), after which the column underwent the cleaning steps to ensure clean elution of sample material from it. This was followed by equilibration steps, where the flow rate returned to match the initial condition (0.15 ml/min) and mobile phase to 80/20 A/B to equilibrate the column for the next analysis. The total cycle time of the method was 14.25 min. Eluate was directed to the mass spectrometer via ZSpray™ atmospheric pressure electrospray ionization source operating in the positive ion mode. Total Ion Count of PYY_1‐36_ and PYY_3‐36_ were measured in multiple reaction monitoring (MRM) mode optimised for each compound. Summed transitions of each peptide were used for quantification. MRM and MS parameters for quantification of PYY compounds can be found in Table 1. Examples of chromatograms are shown in Supporting information: Figure 1. Ions with specific *m/z* for each one of the four analysed compounds were determined and quantified by peak area ratios against the calibration curve, which were constructed using a linear regression with 1/x^2^ weighting factor excluding the origin.

**Table 1:** Details of MRM transitions used for quantification of PYY compounds and corresponding internal standards (IS). Dwell time was set to 0.222 s for all transitions.

Analyte	Ion type	Retention time (min)	Precursor *m*/*z*	Product *m*/*z*	Cone voltage (V)	Collision energy (eV)
PYY_1‐36_	[M + H]^7+^	6.62	616.73	739.45	60	15
	[M + H]^6+^	6.59	719.32	703.09	60	20
PYY_1‐36_ IS	[M + H]^7+^	6.60	620.63	746.82	60	15
	[M + H]^6+^	6.60	723.93	708.47	60	20
PYY_3‐36_	[M + H]^6+^	6.36	675.93	739.39	60	18
	[M + H]^7+^	6.36	579.48	591.90	60	15
PYY_3‐36_ IS	[M + H]^6+^	6.34	680.60	746.11	60	18
	[M + H]^7+^	6.37	583.51	597.35	60	15

*Note*: Dwell time was set to 0.222 s for all transitions.

Abbreviations: MRM, multiple reaction monitoring; PYY, peptide tyrosine tyrosine.

**Figure 1:**
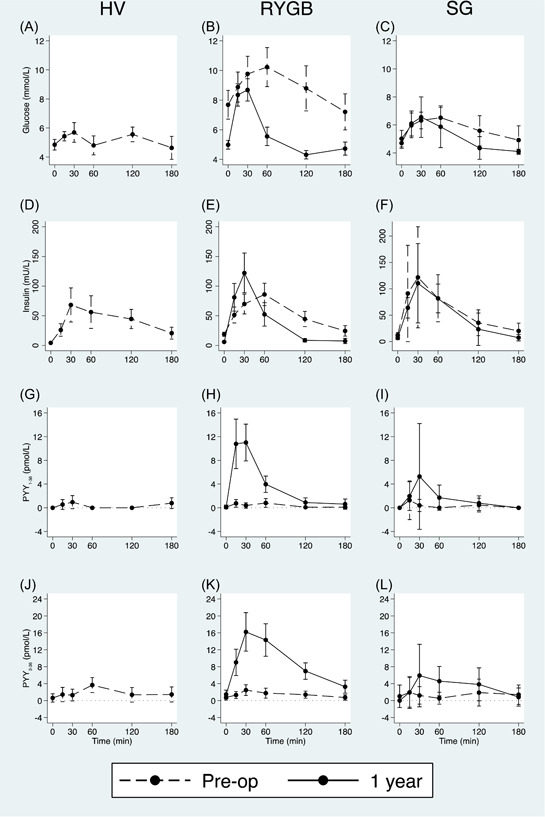
Roux‐en‐Y gastric bypass (RYGB) is associated with an enhanced postprandial peptide tyrosine tyrosine (PYY) response, unlike sleeve gastrectomy (SG). Mean and 95% confidence interval (error bars) plotted against time after mixed meal on x‐axis for glucose (A, B, C), insulin (D, E, F), PYY_1‐36_ (G, H, I), PYY_3‐36_ (J, K, L) during a mixed‐meal test before surgery and 1 year after bariatric surgery. (A, D, G, J) show response in healthy volunteers (HV): baseline only. (B, E, H, K) show response to RYGB at baseline and 1 year. (C, F, I, L) show response to SG at baseline and 1 year. [Color figure can be viewed at wileyonlinelibrary.com]

### 2.6 Assay characteristics

The assay validation was carried out according to the recommendations of Guideline on bioanalytical method validation from Clinical and Laboratory Standards Institute (CLSI – C62‐A), European Medicines Agency (EMA) and the U.S. Department of Health and Human Services Food and Drug Administration (FDA).^19^, ^20^, ^21^ The interassay mean CV was <15%, with CV% ranging from 11.5% to 12.0% for PYY_1‐36_, and 12.5% to 15.1% for PYY_3‐36_ across three levels of QCs, 20 replicates for each level on 11 separate occasions. The intra‐assay imprecision ranged from 10.8% to 14.6% for PYY_1‐36_ and from 8.4% to 14.8% for PYY_3‐36_ with mean of CVs <13.0% for both compounds. Intraassay precision was obtained from the analysis of 11 replicates for each of 3 level QCs in a single analytical run (Supporting Information: Table 1). The LLOQ for PYY_1‐36_ and PYY_3‐36_ by LC‐MS/MS assay were set at 2.44 and 2.04 pmol/L, respectively, with CV < 20%, S/N ratio >10, and an instrument response >5 times greater than the blank, 20BMA (Supporting Information: Table 4). The method was linear up to 100 pmol/L and a dilution series demonstrated good linearity and suitability of 20BMA as a diluent for the plasma samples with concentrations of PYY above 100 pmol/L (Supporting Information: Figure 2).

The mean extraction recovery of spiked human plasma samples for PYY_1‐36_ was 85.2% and for PYY_3‐36_ 92.1%. This was deemed acceptable given that an adequate analytical sensitivity to detect clinically appropriate concentrations of each analyte was achieved. The extraction recovery of spiked surrogate matrix (20BMA) and postextraction spiked human deactivated plasma yielded mean values of 108.2% for PYY_1‐36_ and 85.4% for PYY_3‐36_ (Supporting Information: Table 2). This assured consistent extraction recovery even when different matrices were used.

Matrix effect experiments showed that ion suppression was adequately compensated for by internal standards, measured at three concentrations for both species. Mean matrix effect for EDTA plasma was 99.6% for PYY_1‐36_ and 91.2% for PYY_3‐36_ (Supporting Information: Table 3).

There was no significant carry‐over observed for each analyte (Supporting Information: Figure 3).

Specificity and potential interferences were determined by measuring 50 pmol/L PYY_1‐36_ and PYY_3‐36_ spiked samples in the presence of 100 pmol/L of PYY_1‐34_, PYY_3‐34_, NPY and PP, indicating that the assay demonstrates sufficient specificity as it can distinguish precisely between these closely related peptides, with a minimal molecular weight difference of only 28 Da between the peptides and the stable isotope labelled internal standards. No interference and no changes to the analyte target values were detected (Supporting Information: Figure 4).

The plasma stored aliquots were stable for both compounds for two freeze–thaw cycles and extracts were stable at 4°C for up to 5 days (with < 20% change from baseline).

### 2.7 Data analysis and statistical methods

Data acquisition and analyses including calibration curves were carried out using Waters TargetLynx V4.1 software. The homoeostatic model assessment percentage insulin sensitivity (HOMA2%S) was calculated using the iHOMA2 software, using default settings.^22^ Other statistical analyses were carried out using STATA 15.1 (STATACorp LLC). The distribution of parameters was assessed using kernel density plots and *Q*‐*Q* plots versus idealised normal distributions. A repeated‐measures linear mixed model was used for analysis of glucose, insulin and PYY concentrations as well as the area‐under‐concentration curve (AUC) analysis. GraphPad Prism 9.0.2 (GraphPad Software) was used for the calculation of AUC values using the trapezoid method. For the purposes of analysis, analyte concentrations smaller than the LLOQ were set at zero.

## 3 RESULTS

### 3.1 PYY_1‐36_ and PYY_3‐36_ secretion in response to MMT is similar between nonobese healthy volunteers and preoperative obese patients

It has been reported that obese patients have lower fasting plasma PYY concentrations and a reduced secretion of PYY after eating.^8^ We used the LC‐MS/MS assay to study PYY dynamics after an MMT stimulus in nonobese healthy volunteers (HV: *n* = 10, mean age 39.4 years, mean BMI 24.6 kg/m^2^, none diabetic) and compared these to the baseline postprandial responses in our surgical patients (RYGB: *n* = 24, mean age 47.9 years, mean BMI 43.1 kg/m^2^, 62.5% diabetic; SG: *n* = 6, age 43.2 years, BMI 46.5 kg/m^2^, none diabetic). Although there were clear differences in HbA1c between the HV and RYGB groups, and in insulin sensitivity as estimated by HOMA2%S between HV and both surgical groups, there were no significant differences between HV and both surgical groups in terms of fasting and postprandial secretion of both PYY_1‐36_ and PYY_3‐36_ (Figure 1G,J; Table 2). Indeed, we found no significant correlation of fasting PYY_1‐36_, fasting PYY_3‐36_, total PYY_1‐36_ and PYY_3‐36_ AUC, PYY_1‐36_ and PYY_3‐36_ C_max_ to BMI across all participants (HV, RYGB, SG) at baseline (data not shown), suggesting that BMI is not influential on fasting and post‐prandial PYY secretion.

**Table 2:** Clinical characteristics and dynamics of glucose, insulin, peptide YY in healthy non‐obese volunteers, and in obese patients before and after bariatric surgery.

Parameter	Tpt	HV (*n* = 10)	*p* value Surgical groups vs HV	Surgery (*n* = 30)	RYGB (*n* = 24)	SG (n = 6)	*p* value between surgical groups
Gender	Base	7 F: 3 M	*p* = .447*	22 F: 6 M	16 F: 4 M	6 F: 2 M	*p* = .603*
Diabetic *yes/no*	Base	0/10 (0%)	*p* < .001*	15/30 (50%)	15/24 (62.5%)	0/8 (0%)	*p* = .017*

		Est. marginal mean [95% CI]	Contrast [95% CI] of surgical group vs HV		Estimated marginal means [95% CI]		
Parameter	Tpt	HV (*n* = 10)	RYGB	SG	Surgery (*n* = 30)	RYGB (*n* = 24)	SG (*n* = 6)
**Clinical**							
Age years	Base	39.4 [31.2, 47.6]	9.7 [−0.02, 19.5]	3.8 [−9.6, 17.1]	47.9 [43.4, 52.4]	49.1 [44.1, 54.1]	43.2 [33.1, 53.2]
			*p* = .050	*p* = .581			
BMI kg/m^*2*^	Base	24.6 [21.1, 28.0]	18.5 [14.4, 22.6]	21.9 [16.4, 27.5]	43.7 [41.8, 45.7]	43.1 [40.9, 45.3]	46.5 [42.1, 50.9]
			*p* < .001	*p* < .001			
	1 year	N/A	N/A	N/A	30.8 [28.9, 32.8]	29.9 [27.7, 32.1]	34.8 [30.4, 39.2]
TWL *%*	1 year	N/A	N/A	N/A	−29.4 [−31.8, −26.9]	−30.5 [−33.2, −27.7]	−24.9 [−30.4, −19.4]
HbA1c mmol/mol	Base	32.3 [25.2, 39.4]	21.1 [12.6, 29.5]	8.7 [−2.9, 20.3]	50.9 [47.3, 54.5]	53.4 [49.4, 57.4]	41.0 [33.0, 49.0]
			*p* < .001	*p* = .140			
	1 year	N/A	N/A	N/A	36.5 [32.9, 40.1]	36.4 [32.4, 40.4]	37.0 [29.0, 45.0]
HOMA2%S	Base	219.7 [192.9, 246.5]	−168 [−199, −136]	−139 [−183, −95]	57.8 [37.5, 78.1]	52.2 [29.5, 74.8]	80.5 [35.1, 125.9]
			*p* < .001	*p* < .001			
	1 year	N/A	N/A	N/A	151.4 [131.1, 171.7]	156.1 [133.4, 178.8]	132.3 [86.9, 177.7]
**Glucose**							
Fasting mmol/L	Base	4.9 [3.9, 5.8]	2.8 [1.6, 3.9]	0.1 [−1.5, 1.7]	7.1 [6.6, 7.6]	7.6 [7.1, 8.2]	5.0 [3.9, 6.0]
			*p* < .001	*p* = .901			
	1 year	N/A	N/A	N/A	4.9 [4.4, 5.4]	5.0 [4.5, 5.5]	4.6 [3.5, 5.6]
*C*_max_ mmol/L	Base	6.0 [4.5, 7.6]	4.7 [2.9, 6.5]	0.9 [−1.6, 3.4]	10.0 [9.2, 10.8]	10.7 [9.8, 11.7]	6.9 [5.1, 8.8]
			*p* < .001	*p* = .490			
	1 year	N/A	N/A	N/A	8.9 [8.1, 9,7]	9.4 [8.5, 10.4]	6.8 [5.0, 8.7]
tAUC_0‐180_ mmol·min/L	Base	934 [683, 1185]	680 [381, 979]	111 [−299, 521]	1501 [1380, 1621]	1614 [1480, 1749]	1045 [776,1314]
			*p* < .001	*p* = .596			
	1 year	N/A	N/A	N/A	991 [871, 1112]	1009 [874, 1143]	922 [653, 1191]
**Insulin**							
Fasting mU/L	Base	4.3 [0.4, 8.2]	14.6 [9.9, 19.2]	9.1 [2.8, 15.5]	17.8 [15.7, 19.8]	18.8 [16.6, 21.1]	13.4 [8.9, 17.9]
			*p* < .001	*p* = .005			
	1 year	N/A	N/A	N/A	6.7 [4.7, 8.7]	6.3 [4.0, 8.5]	8.5 [3.9, 13.0]
*C*_max_ mU/L	Base	73.7 [44.3, 103.1]	18.7 [−16.3, 53.7]	59.2 [11.2, 107.2]	100.5 [78.3, 122.7]	92.4 [67.6, 117.2]	133.0 [83.3, 182.6]
			*p* = .246	*p* = .016			
	1 year	N/A	N/A	N/A	126.9 [104.7, 149.0]	128.7 [103.9, 153.6]	119.3 [69.7, 168.9]
tAUC_0‐180_ mU·min/L	Base	7789 [5065, 10512]	1973 [−1269, 5215]	2844 [−1604, 7291]	9936 [8361, 11509]	9762 [8002, 11522]	10632 [7112, 14152]
			*p* = .237	*p* = .210			
	1 year	N/A	N/A	N/A	7449 [5875, 9023]	7110 [5350, 8870]	8805 [5284, 12325]
**PYY**_**1‐36**_							
Fasting pmol/L	Base	0.00 [−0.20, 0.20]	0.09 [−0.16, 0.33]	0.00 [−0.32, 0.32]	0.07 [−0.12, 0.26]	0.09 [−0.13, 0.30]	0.00 [−0.43, 0.43]
			*p* = .475	*p* = 1.000			
	1 year	N/A	N/A	N/A	0.12 [−0.07, 0.32]	0.15 [−0.06, 0.37]	0.00 [−0.43, 0.43]
*C*_max_ pmol/L	Base	2.37 [1.24, 3.50]	−0.85 [−2.20, 0.50]	−0.65 [−2.50, 1.20]	1.56 [−0.76, 3.88]	1.52 [−1.07, 4.11]	1.72 [−3.47, 6.90]
			*p* = .218	*p* = .489			
	1 year	N/A	N/A	N/A	12.75 [10.43, 15.07]	14.43 [11.84, 17.02]	6.05 [0.87, 11.23]
tAUC_0‐180_ pmol·min/L	Base	211 [146, 276]	−69 [−146, 9]	−81 [−187, 26]	140 [54.7, 226]	143 [47, 238]	131 [−61, 322]
			*p* = .083	*p* = .137			
	1 year	N/A	N/A	N/A	659 [574, 744]	733 [637, 829]	363 [172, 554]
**PYY**_**3‐36**_							
Fasting pmol/L	Base	0.63 [−0.20, 1.46]	0.13 [−0.86, 1.12]	−0.63 [−1.99, 0.73]	0.61 [−0.08, 1.30]	0.76 [−0.01, 1.54]	0.00 [−1.53, 1.53]
			*p* = .793	*p* = 0.362			
	1 year	N/A	N/A	N/A	1.36 [0.67, 2.06]	1.45 [0.67, 2.22]	1.03 [−0.52, 2.59]
*C*_max_ pmol/L	Base	4.40 [2.71, 6.09]	−1.00 [−3.00, 1.02]	−1.13 [−3.89, 1.63]	3.38 [0.97, 5.78]	3.40 [0.71, 6.10]	3.27 [−2.11, 8.65]
			*p* = .332	*p* = .421			
	1 year	N/A	N/A	N/A	16.37 [13.97, 18.78]	18.77 [16.08, 21.46]	6.78 [1.40, 12.17]
tAUC_0‐180_ pmol·min/L	Base	483 [321, 646]	−128 [−322, 65]	−136 [−402, 130]	354 [137, 570]	355 [114, 597]	347 [−136, 831]
			*p* = .194	*p* = .316			
	1 year	N/A	N/A	N/A	1514 [1298, 1730]	1715 [1473, 1956]	711 [228, 1194]
**PYY**_**1‐36**_ **:PYY**_**3‐36**_ **ratio**							
C_max_	Base	0.71 [0.47, 0.94]	−0.46 [−0.74, −0.17]	−0.48 [−0.87, −0.09]	0.25 [0.11, 0.39]	0.26 [0.10, 0.41]	0.22 [−0.08, 0.52]
			*p* = .002	*p* = .016			
	1 year	N/A	N/A	N/A	0.78 [0.65, 0.92]	0.82 [0.67, 0.97]	0.65 [0.35, 0.95]

	Contrast [95% CI] of SG vs RYGB		Contrast (95% CI] of 1 yr vs Baseline	
Parameter	Baseline	1 year	RYGB	SG
**Clinical**				
Age years	−6.0 [−17.2, 5.3]	N/A	N/A	N/A
	*p* = .298			
BMI kg/m^*2*^	3.4 [−1.5, 8.3]	5.0 [0.0, 9.9]	−13.2 [−14.7, −11.7]	−11.7 [−14.6, −8.7]
	*p* = .172	*p* = .048	*p* < .001	*p* < .001
TWL *%*	N/A	5.6 [−0.6, 11.7]	N/A	N/A
		*p* = .076		
HbA1c mmol/mol	−12.4 [−21.3, −3.5]	0.6 [−8.3, 9.5]	−17.0 [−22.3, −11.7]	−4.0 [−14.6, 6.6]
	*p* = .007	*p* = .891	*p* < .001	*p* = .459
HOMA2%S	28.3 [−22.4, 79.1]	−23.8 [−74.6, 26.9]	104.0 [85.0, 123.0]	51.8 [13.8, 89.8]
	*p* = .274	*p* = .357	*p* < .001	*p* = .008
**Glucose**				
Fasting mmol/L	−2.7 [−3.9, −1.5]	−0.4 [−1.6, 0.8]	−2.6 [−3.4, −1.9]	−0.4 [−1.8, 1.0]
	*p* < .001	*p* = .486	*p* < .001	*p* = .598
*C*_max_ mmol/L	−3.8 [−5.9, −1.8]	−2.6 [−4.7, −0.5]	−1.3 [−2.4, −0.3]	−0.1 [−2.2, 2.0]
	*p* < .001	*p* = .014	*p* = .015	*p* = .927
tAUC_0‐180_ mmol·min/L	−569 [−870, −268]	−86 [−387, 215]	−606 [−787, −425]	−123 [−484, 238]
	*p* < .001	*p* = .574	*p* < .001	*p* = .505
**Insulin**				
Fasting mU/L	−5.4 [−10.5, −0.4]	2.2 [−2.9, 7.3]	−12.6 [−15.1, −10.1]	−5.0 [−9.9, −0.005]
	*p* = .035	*p* = .394	*p* < .001	*p* = .049
*C*_max_ mU/L	40.5 [−15.0, 96.0]	−9.4 [−64.9, 46.0]	36.3 [7.1, 65.5]	−13.6 [−72.0, 44.7]
	*p* = .152	*p* = .734	*p* = .015	*p* = .647
tAUC_0‐180_ mU·min/L	870 [−3065, 4806]	1695 [−2241, 5630]	−2652 [−4437, −867]	−1828 [−5397, 1742]
	*p* = .665	*p* = .399	*p* = .004	*p* = .316
**PYY**_**1‐36**_				
Fasting *pmol/L*	−0.09 [−0.57 0.39]	−0.15 [−0.64, 0.33]	0.07 [−0.24, 0.37]	0.00 [−0.61, 0.61]
	*p* = .721	*p* = .530	*p* = .668	*p* = 1.000
*C*_max_ pmol/L	0.20 [−5.60, 5.99]	−8.38 [−14.18, −2.59]	12.91 [9.56, 16.25]	4.33 [−2.36, 11.02]
	*p* = .947	*p* = .005	*p* < .001	*p* = .204
tAUC_0‐180_ pmol·min/L	−12 [−226, 202]	−370 [−584, −156]	590 [457, 723]	233 [−33, 499]
	*p* = .912	*p* < .001	*p* < .001	*p* = .087
**PYY**_**3‐36**_				
Fasting pmol/L	−0.76 [−2.50, 0.97]	−0.41 [−2.14, 1.32]	0.68 [−0.41, 1.78]	1.03 [−1.16, 3.23]
	*p* = .389	*p* = .641	*p* = .223	*p* = .356
C_max_ pmol/L	−0.14 [−6.16, 5.88]	−11.99 [−18.00, −5.97]	15.37 [12.04, 18.70]	3.52 [−3.14, 10.18]
	*p* = .964	*p* < .001	*p* < .001	*p* = .301
tAUC_0‐180_ pmol·min/L	−8 [−548, 533]	−1004 [−1544, −463]	1359 [1068, 1651]	364 [−220, 947]
	*p* = .977	*p* < .001	*p* < .001	*p* = .222
**PYY**_**1‐36**_ **:PYY**_**3‐36**_ **ratio**				
C_max_	−0.03 [−0.37, 0.30]	−0.17 [−0.51, 0.16]	0.56 [0.36, 0.76]	0.43 [0.04, 0.81]
	*p* = .845	*p* = .317	*p* < .001	*p* = .03

*Note*: Fasting concentrations, Cmax (maximal concentration during mixed meal test), and total area‐under‐curve from 0 to 180 min (tAUC_0‐180_) for glucose, insulin, GLP‐1, PYY_1‐36_ and PYY_3‐36_ shown during mixed meal test at baseline and 1 year. For insulin in pmol/L, multiply by 6. Estimated marginal means [95% CI] shown for fasting, Cmax, total AUC_0‐180_ for all patients, and for RYGB and SG separately. Data analysed using Fisher exact test for categorical variables*, or repeated measures linear mixed model for continuous variables as indicated. Contrasts of marginal linear predictions with 95% CI shown for HV versus surgical groups at baseline, for SG vs RYGB at baseline and 1 year timepoints, as well as for baseline versus 1 year for RYGB and SG groups.

Abbreviations: Base, baseline; HV, healthy volunteers; HOMA2%S,  estimated percentage insulin sensitivity using interactive, 24‐variable homoeostatic model assessment 2 on default settings22; F, female; M, male; N/A, not applicable; RYGB, Roux‐en‐Y gastric bypass; SG, sleeve gastrectomy; SD, standard deviation; Tpt, timepoint; TWL, total weight loss.

### 3.2 Postprandial secretion of PYY_1‐36_ and PYY_3‐36_ is markedly amplified after RYGB but not after SG

We used the LC‐MS/MS assay to study the dynamics of PYY secretion in our obese patients at two timepoints, before and at 1 year after bariatric surgery. Thirty participants (73% female) were prospectively evaluated with a mean BMI of 43.7 kg/m^2^ at baseline and HbA1c of 50.9 mmol/mol (Table 2). Six participants underwent SG and 24 RYGB. Of note, none of the SG participants had type 2 diabetes whereas 62.5% of the RYGB participants had diabetes (*p* = .017, Fisher exact test). No significant difference between surgical type in baseline HOMA2%S (*p* = .274) was noted but there was a significant difference in baseline HbA1c, which was lower in the SG group compared with RYGB (mean contrast −12.4 mmol/mol, *p* = .007), consistent with the differences in diabetes status. As expected, there was a significant total weight loss after 1 year of −29.4%, which was not statistically significantly different between the RYGB (−30.5%) and SG groups (−24.9%, mean contrast 5.6%, *p* = .076). This weight loss was accompanied by significant improvements in HbA1c in the RYGB group (−17.0 mmol/mol, *p* < .001) but not in the SG group (−4.0 mmol/mol, *p* = .459) reflecting the limited scope for improvement in glycaemia in the nondiabetic SG group. Both RYGB and SG, however, saw significant improvements in HOMA2%S at 1 year compared with baseline (*p* < .001, *p* = .008, respectively).

Consistent with the differences in diabetes status, the RYGB group had a higher baseline fasting glucose and poorer glucose tolerance (as assessed by total glucose AUC_0‐180_) than SG (*p* < .001 for both parameters; Table 2, Figure 1B,C). There were significant improvements in fasting glucose and total glucose AUC_0‐180_ after RYGB (*p* < .001 for both parameters) but not after SG (*p* = .598 and 0.505, respectively), again reflecting the limited scope for improvement in glycaemia in the nondiabetic SG group. At baseline, fasting insulin was higher in the RYGB group compared with SG (*p* = .035; Table 2, Figure 1E,F), but after both types of surgery this fell significantly (*p* < .001 and *p* = .049). One year after RYGB, there were significant increases in insulin C_max_ and total insulin AUC_0‐180_ over baseline (*p* = .015 and .004 for each parameter) but not for SG (*p* = .647, 0.316 respectively).

At preoperative baseline, the PYY_1‐36_ and PYY_3‐36_ concentrations as assessed by LC‐MS/MS were low and consumption of the MMT led to no or little postprandial stimulation (Figure 1H, I, K, L). At baseline, there were no differences between RYGB and SG in C_max_ for PYY_1‐36_ (*p* = .721), in C_max_ for PYY_3‐36_ (*p* = .964), in PYY_1‐36_ AUC_0‐180_ (*p* = .912) nor in PYY_3‐36_ AUC_0‐180_ (*p* = .977).

### 3.3 Postprandial PYY_1‐36_ and PYY_3‐36_ secretion after SG is diminished in comparison to RYGB at 1 year

One year postoperatively, fasting PYY_1‐36_ and PYY_3‐36_ did not significantly differ from baseline for both types of surgery, but the MMT stimulated marked postprandial secretion of both species of PYY in RYGB (Table 2; Figure 1H,K). In comparison between baseline to 1 year for RYGB, there were significant increases in *C*_max_ for PYY_1‐36_ (*p* < .001), in PYY_1‐36_ AUC_0‐180_ (*p* = .087), in *C*_max_ for PYY_3‐36_ (*p* < .001), and in total PYY_3‐36_ AUC_0‐180_ (*p* < .001). Although there was a numerical increase in PYY secretion postprandially for SG comparing baseline to 1 year (Figure 1I,L), these were not statistically significant: *C*_max_ for PYY_1‐36_ (*p* = .204), PYY_1‐36_ AUC_0‐180_ (*p* = .087), *C*_max_ for PYY_3‐36_ (*p* = .301), and total PYY_3‐36_ AUC_0‐180_ (*p* = .222). At 1 year after surgery, there was overall greater postprandial secretion of both species of PYY in RYGB compared with SG with significant differences between the surgical types in *C*_max_ for PYY_1‐36_ (*p* = .005), in *C*_max_ for PYY_3‐36_ (*p* < .001), in PYY_1‐36_ AUC_0‐180_ (*p* < .001) and in total PYY_3‐36_ AUC_0‐180_ (*p* < .001).

### 3.4 DPP‐4 activity is increased in obese patients and this reduces after bariatric surgery

DPP‐4, the enzyme that converts PYY_1‐36_ to PYY_3‐36_, is thought to be a mediator of the link between obesity and diabetes by inactivating incretins such as GLP‐1, and regulating inflammation and insulin resistance in the liver and adipose tissue.^23^ Plasma DPP‐4 activity is reported to be increased in people with diabetes^24^ and obese patients^25^ relative to healthy controls. As an index of DPP‐4 activity, we calculated the ratio of the maximal concentrations during the MMT (C_max_) of PYY_1‐36_ to PYY_3‐36_ in our HV, RYGB, and SG groups. At baseline we found that this ratio was significantly higher in our HV groups (0.71) than in the RYGB group (contrast −0.46, *p* = .002) and the SG group (contrast −0.48, *p* = .016) suggesting an increased conversion of PYY_1‐36_ to PYY_3‐36_ and hence DPP‐4 activity in the obese patients relative to the healthy volunteers. One year after surgery, there were significant increases in the PYY_1‐36_:PYY_3‐36_ C_max_ ratio (RYGB: + 0.56, *p* < .001; SG: + 0.43, *p* = .03) implying that DPP‐4 activity was reduced 1 year after surgery.

## 4 DISCUSSION

Bariatric surgery's efficacy in inducing sustainable weight loss and its metabolic benefits for people with diabetes have been well described.^26^ The marked increase of postprandial gut hormone secretion is thought to be one of the main mechanisms mediating the effects of surgery in weight reduction and glucose homoeostasis. This phenomenon has been most well characterised for proglucagon peptides such as GLP‐1, oxyntomodulin, and glicentin.^16^, ^27^ Herein, we utilize a validated, ultrasensitive, and quantitative LC‐MS/MS assay for PYY_1‐36_ and PYY_3‐36_ to investigate the postprandial secretion of PYY peptides in obesity and the changes after bariatric surgery. Previous studies utilising various immunoassays have detected fasting total PYY levels of 7–19 pmol/L^28^, ^29^ and peak postprandial responses of 10–20 pmol/L^28^ or 20–35 pmol/L^29^ for comparable calorie intakes. In our healthy nonobese volunteers, the baseline levels of both species of PYY were lower (often below our LLOQ of ~2 pmol/L) and there was a minimal postprandial response to around 3–4 pmol/L; a similar pattern was noted by Reverter‐Branchat et al. in their healthy volunteers.^15^ Furthermore, the secretion of both PYY species (fasting and postprandial) was similar between our healthy nonobese volunteers and the obese patients before surgery. Previous work using a radio‐immunoassay for total PYY had suggested that fasting and postprandial secretion was deficient in obese compared with nonobese volunteers and that there was a negative correlation of fasting PYY to BMI.^8^ We did not find a similar correlation in our data; the reduced secretion of PYY and a defective feedback on appetite regulation may not be as important in driving obesity as previously assumed.

After RYGB, we saw amplified postprandial PYY secretion at 1 year but not with SG where the postprandial PYY secretion, although numerically larger, was not statistically different from baseline. Previous studies have consistently observed highly amplified postprandial total PYY secretion which persists in long‐term follow‐up after RYGB but have reported some discordant results with respect to SG.^29^, ^30^, ^31^ Arakawa et al. observed increases in postprandial total PYY secretion with SG only at 26 weeks and not 52 weeks^29^; similarly, Peterli et al. saw significant increases in total PYY postprandial secretion in their early timepoints but this effect regressed to the baseline by 1 year.^31^ On the other hand, Alamuddin et al. observed a persistent and significant increase in postprandial total PYY secretion above baseline at 6 and 18 months after SG.^30^ Our data support the notion that postprandial PYY secretion may regress towards baseline at 1 year after SG, with the caveat that the numbers undergoing SG in our cohort were relatively small; it remains possible that SG is still associated with a relatively small increase in postprandial PYY secretion.

The increase in postprandial secretion of the PYY peptides was clearly more marked after RYGB in comparison to SG. This observation is consistent with most other studies^29^, ^30^, ^31^, ^32^ who found greater postprandial total PYY secretion after RYGB compared with SG although one study showed similar postprandial total PYY secretion between RYGB and SG.^33^ Although there were baseline differences between our RYGB and SG cohorts, primarily with respect to glycaemia and diabetes status, at 1 year the two cohorts were similar in terms of BMI, HbA1c, and total weight loss.

We also found that DPP‐4 activity, as judged by the relative ratios of PYY_1‐36_ to PYY_3‐36_, was higher preoperatively in our obese patients compared with our healthy volunteers. After bariatric surgery DPP‐4 activity fell, and such a reduction in DPP‐4 activity would be expected to drive beneficial metabolic effects such as reduced degradation of incretins both in circulation and within pancreatic islets, as well as reductions in inflammation and insulin resistance within adipose tissue and the liver.^23^ Although previous studies suggest that bariatric surgery does not seem to affect the abnormally increased plasma DPP‐4 activity,^25^, ^34^ our results may reflect the conversion of PYY_1‐36_ to PYY_3‐36_ via tissue DPP‐4 (e.g., pancreatic, intestine, liver, vascular endothelium) and may be a more sensitive marker of the effects of surgery and weight loss on the physiology of DPP‐4 than plasma enzyme activity. These suggestive but preliminary results will need confirmation in future studies.

Our data allow for the distinction of the roles played by each PYY peptide in these surgical procedures. The long‐term persistence of the amplified postprandial secretion of PYY_3‐36_ after RYGB may be an explanation for the better long‐term weight loss observed with this procedure compared with SG.^35^ Another point of difference between the two bariatric procedures concerns the postsurgical erosion of bone mineral density which is more marked with RYGB than SG.^36^ PYY_1‐36_ secretion, Y1 receptor activation, and suppression of osteoblastic activity is linked to increased bone turnover after bariatric surgery.^37^ We found that the postprandial AUC for PYY_1‐36_ after RYGB was larger than after SG. We hypothesize that the differential secretion of PYY_1‐36_ between the two procedures may be an explanation for this phenomenon.

In summary, we have devised a highly sensitive and specific assay for PYY which is able to distinguish between the two principal active species of this peptide. We show that the fasting and postprandial secretion of PYY_1‐36_ and PYY_3‐36_ is minimal in obesity and does not seem to differ significantly from nonobese healthy volunteers. Furthermore, we have confirmed that PYY_1‐36_ and PYY_3‐36_ postprandial secretion is amplified at 1 year after bariatric surgery with RYGB, but not with SG. The detailed and differentiated roles of PYY's active species in physiological processes such as insulin secretion, appetite regulation, bone metabolism, and DPP‐4 activity (both natively, and when inhibited pharmacologically), as well as pathophysiological processes such as obesity and intestinal disease can now be investigated with sensitive and specific LC‐MS/MS assays.

## AUTHOR CONTRIBUTIONS

Anna M. Kowalka, Joyceline Cuenco, Rosemary E. Clarke, Stephane Camuzeaux, Matthew R. Lewis, Harry J. Whitwell, Maria Gomez Romero, and Tricia M.‐M. Tan contributed to the development and validation of the LC‐MS/MS assay. Sanjay Purkayastha, Ahmed R. Ahmed were the surgeons who performed the bariatric surgery. Tricia M.‐M. Tan, Kleopatra Alexiadou, Ahmed R. Ahmed, Sanjay Purkayastha contributed to the execution of the clinical study. Paul Bech performed the RIA analysis of PYY. James Minnion, Stephen R. Bloom and Tricia M.‐M. Tan designed the clinical study. Bernard Khoo contributed to the statistical analysis of the data. All authors contributed to the writing of the manuscript and approved the final version of the manuscript. Tricia M.‐M. Tan is the guarantor of the manuscript and confirms that all authors have had access to the full data set.

## CONFLICT OF INTEREST

James Minnion, Stephen R. Bloom, Tricia M.‐M. Tan are employees and/or share‐holders in Zihipp Ltd., an Imperial College spin‐out company developing analogues of gut hormones for the treatment of obesity. The remaining authors declare no conflict of interest.

## Supporting information

Supporting information.

## Data Availability

Anonymized data are available from the corresponding author on reasonable request.
