# Supplementary material for: The postprandial secretion of peptide YY1‐36 and 3‐36 in obesity is differentially increased after gastric bypass versus sleeve gastrectomy
Source: Clin Endocrinol (Oxf). 2022 Nov 28;99(3):272–84. doi: 10.1111/cen.14846 (PMC10952770; doi:10.1111/cen.14846)

The post-prandial secretion of Peptide YY_1-36_ and _3-36_ in obesity is differentially increased after gastric bypass versus sleeve gastrectomy

**Supplementary material**

Table of contents

[Supplemental Figure 1 (Total Ion Count Chromatograms) 2](#_Toc75360825)

[Supplemental Table 1 (Precision Data) 6](#_Toc75360826)

[Supplemental Figure 2 (Linearity) 6](#_Toc75360827)

[Supplemental Table 2 and 3 (Matrix Effects and Extraction Recovery) 8](#_Toc75360828)

[Supplemental Table 4 (Limits of Quantification) 9](#_Toc75360829)

[Supplemental Figure 3 (Carry-over Experiment) 10](#_Toc75360830)

[Supplemental Figure 4 (Specificity and Interferences) 12](#_Toc75360835)

[References 15](#_Toc75360836)

***Supplemental Figure 1 (Total Ion Count Chromatograms)***

Figure 1 shows the Total Ion Count (TIC) chromatograms of PYY_1-36_ and PYY_3-36_. Two transitions for each compound were combined and used for quantifying both compounds.

|  | PYY_1-36_ | PYY_3-36_ |
| --- | --- | --- |
| TIC for extracted Blank sample spiked with IS | 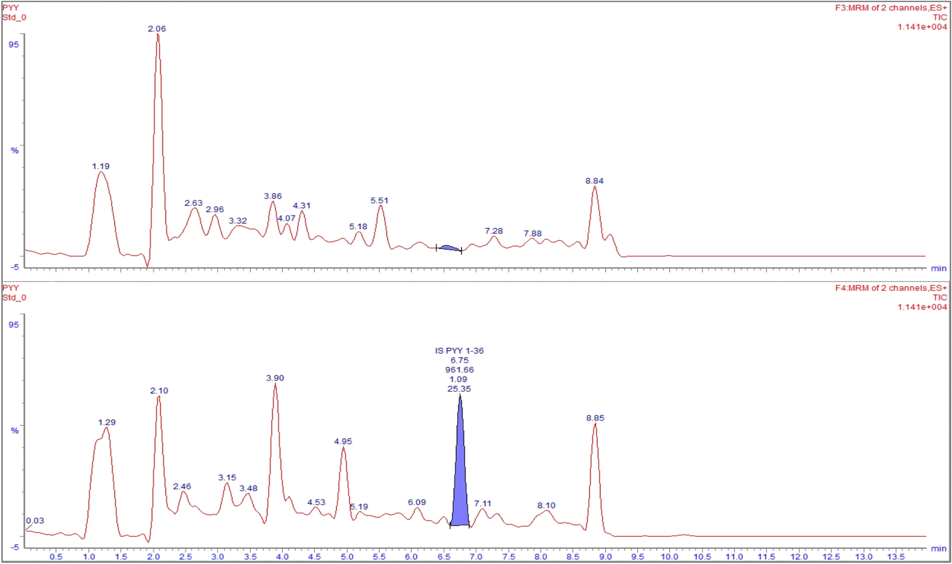  **1a**  **1A** | 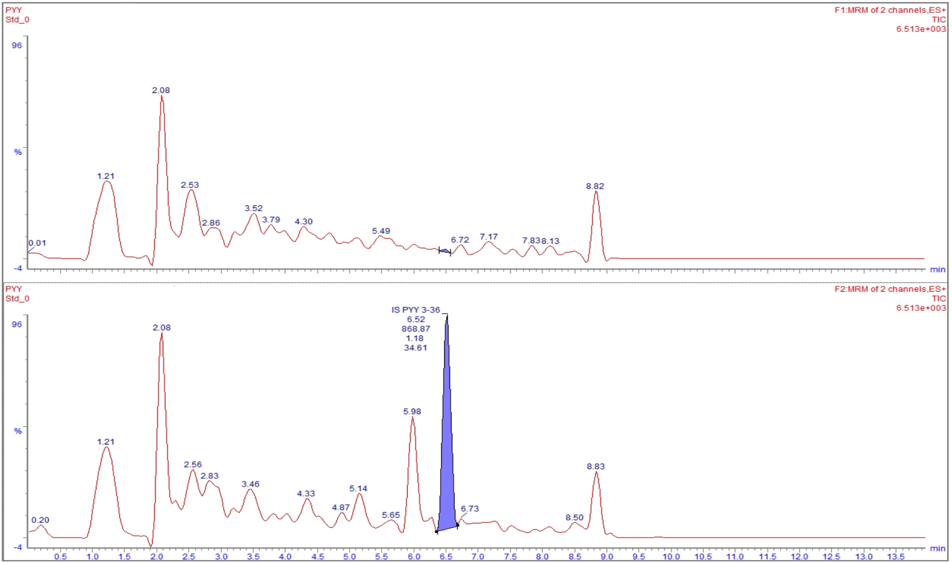  **2a**  **2A** |
| IS spiked calibration standard at the level of LLOQ | 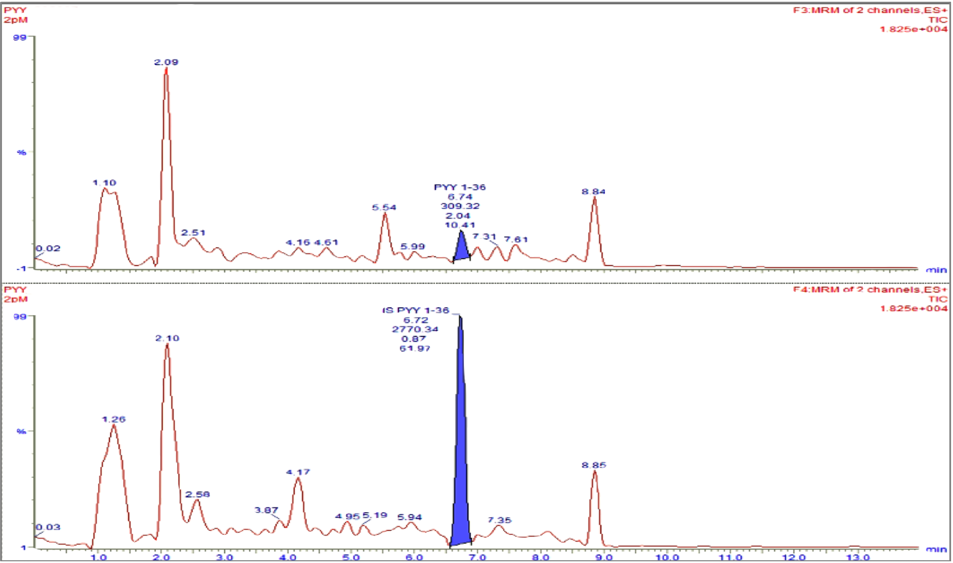  **1b**  **1B** | 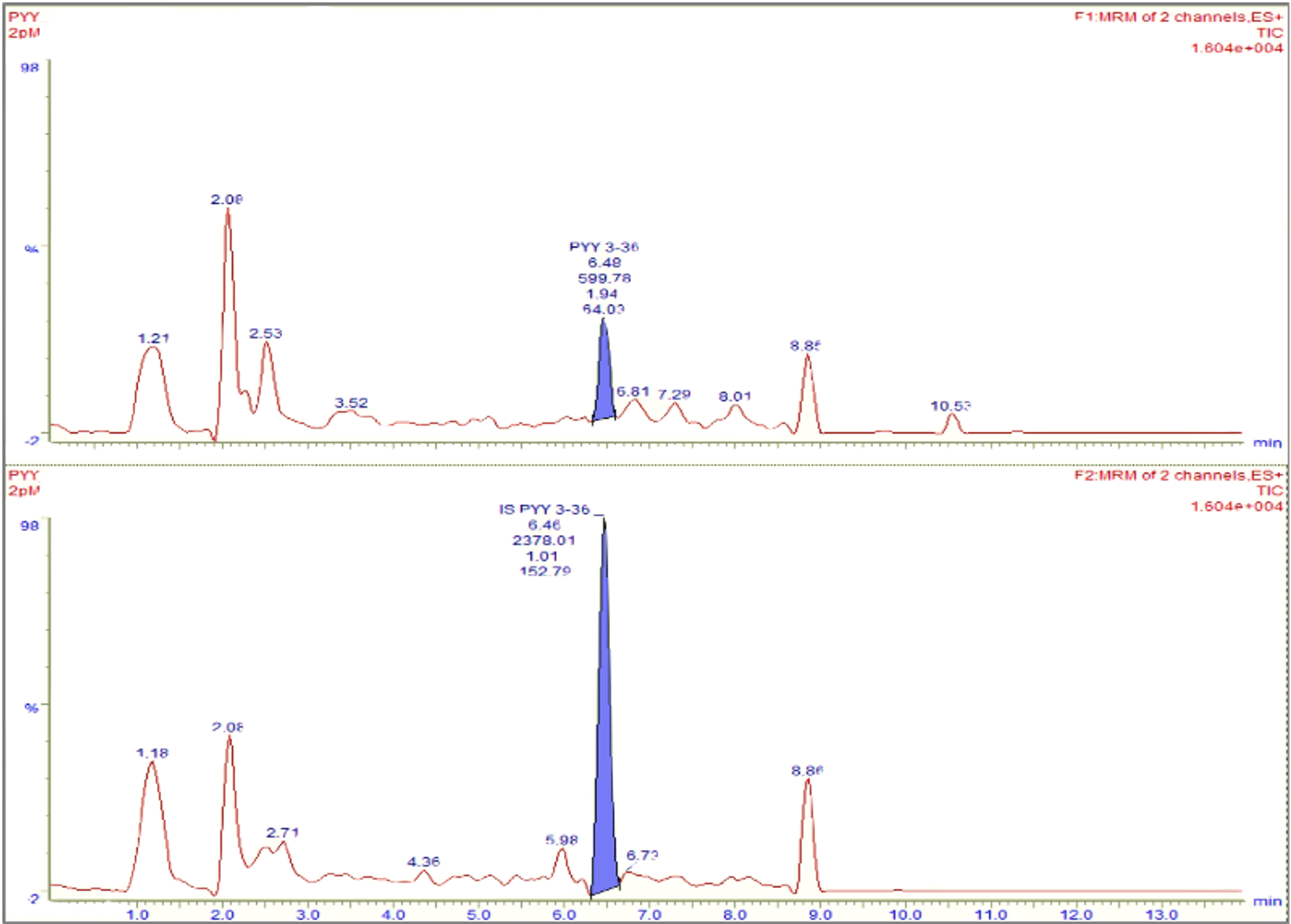  **2b**  **2B** |
| 10 pM standard extracted from spiked plasma zero’ | 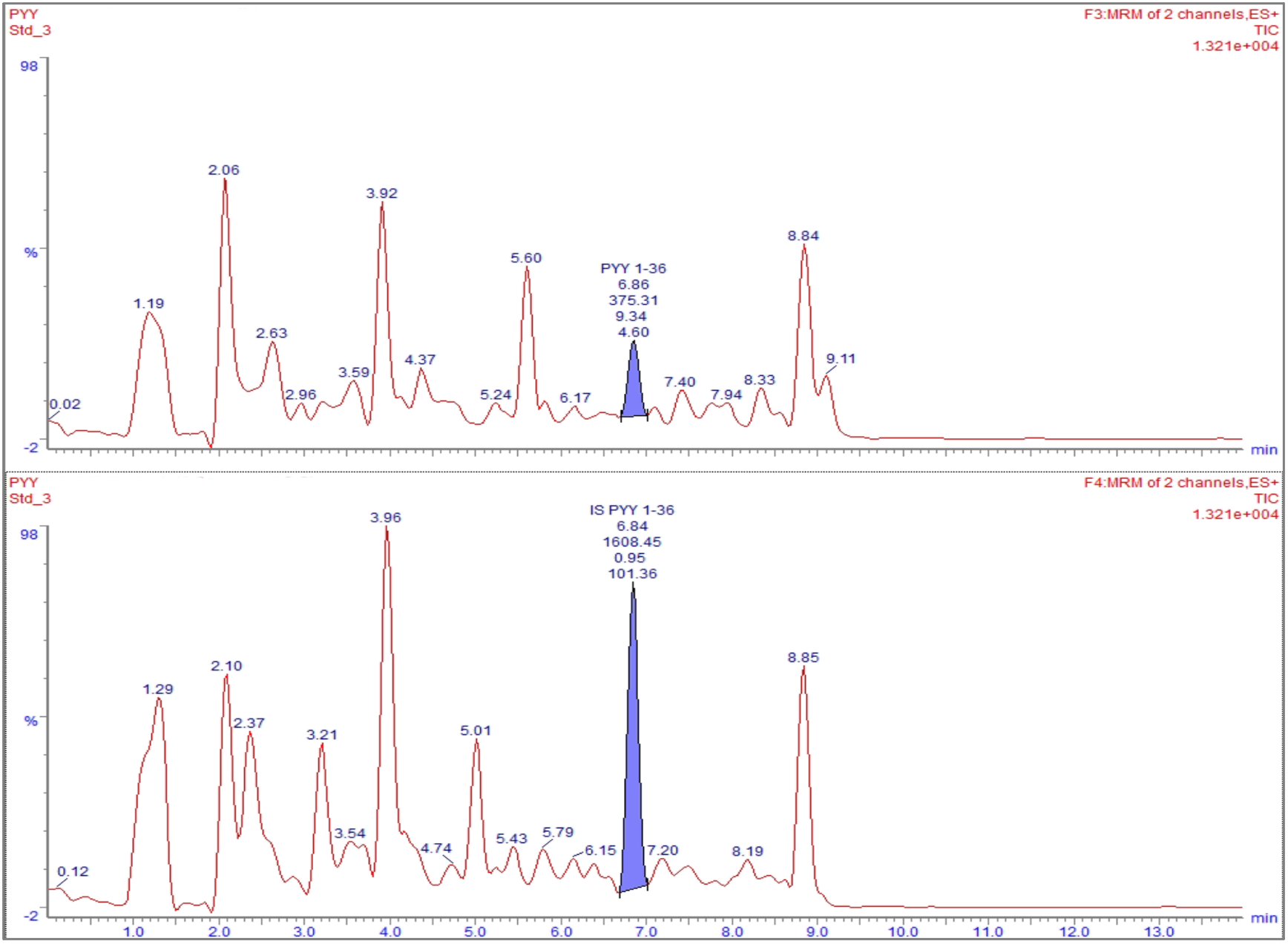  **1c**  **1C** | 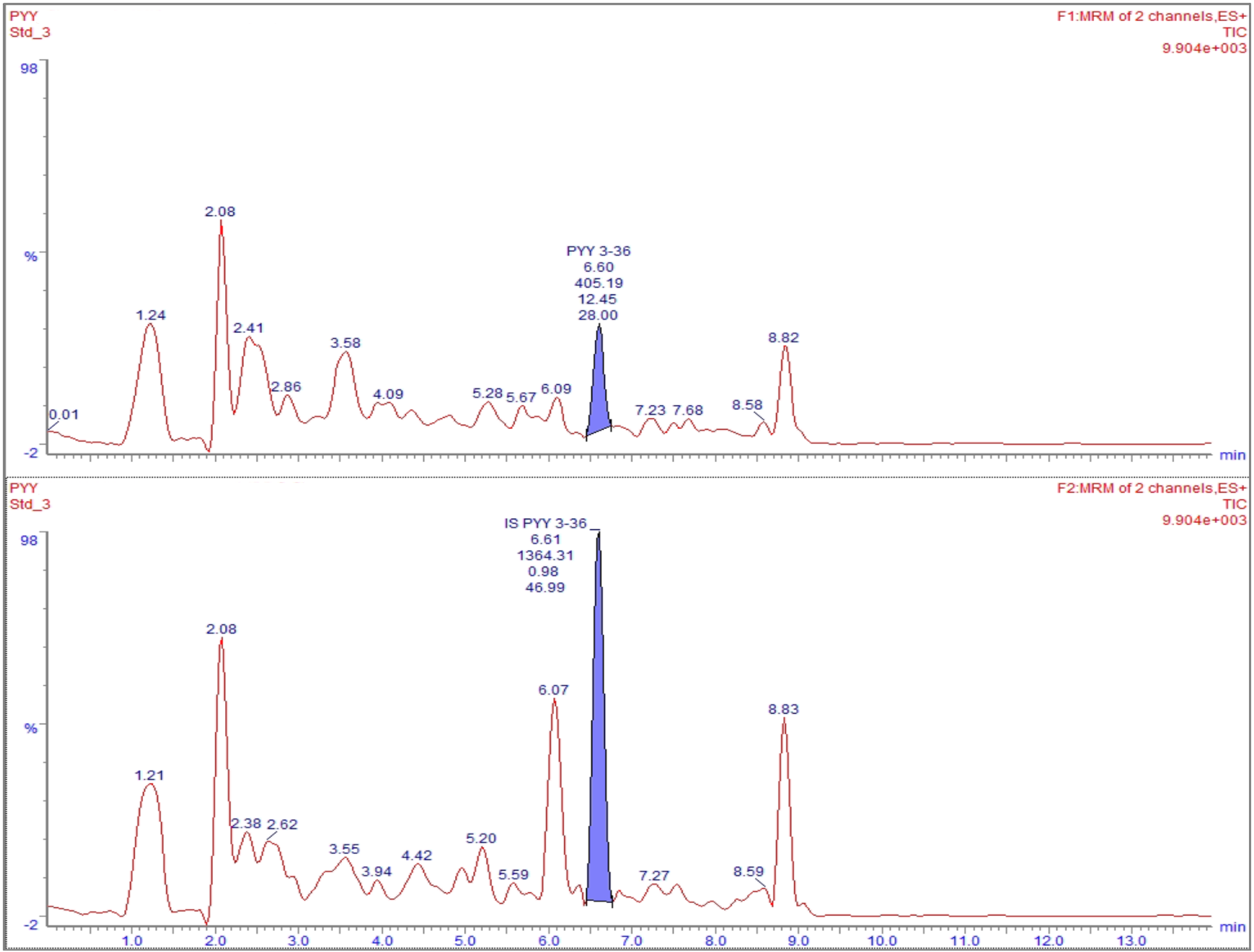  **2c**  **2C** |
| 10 pM standard extracted from spiked 20BMA | 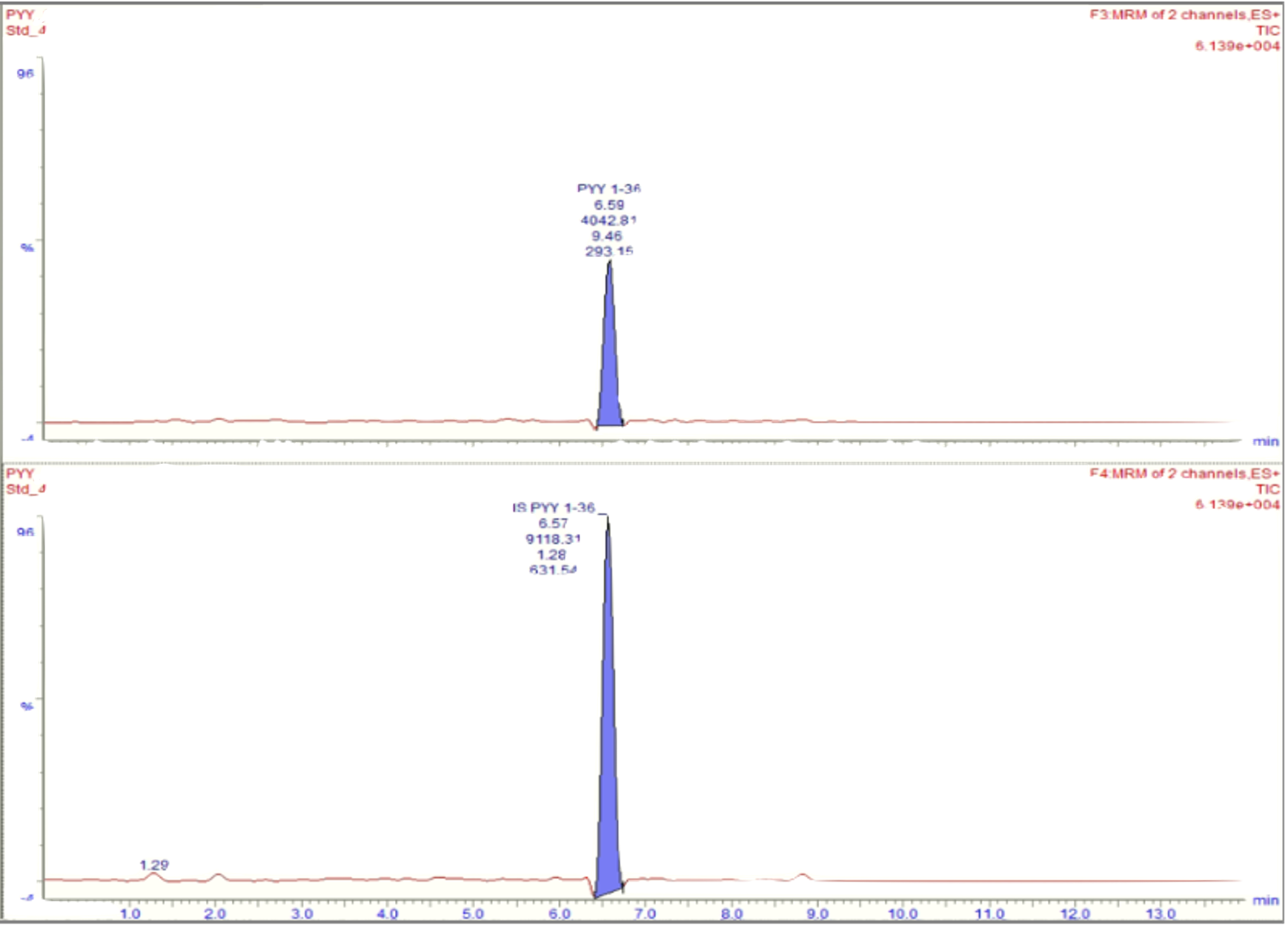  **1d**  **1D** | 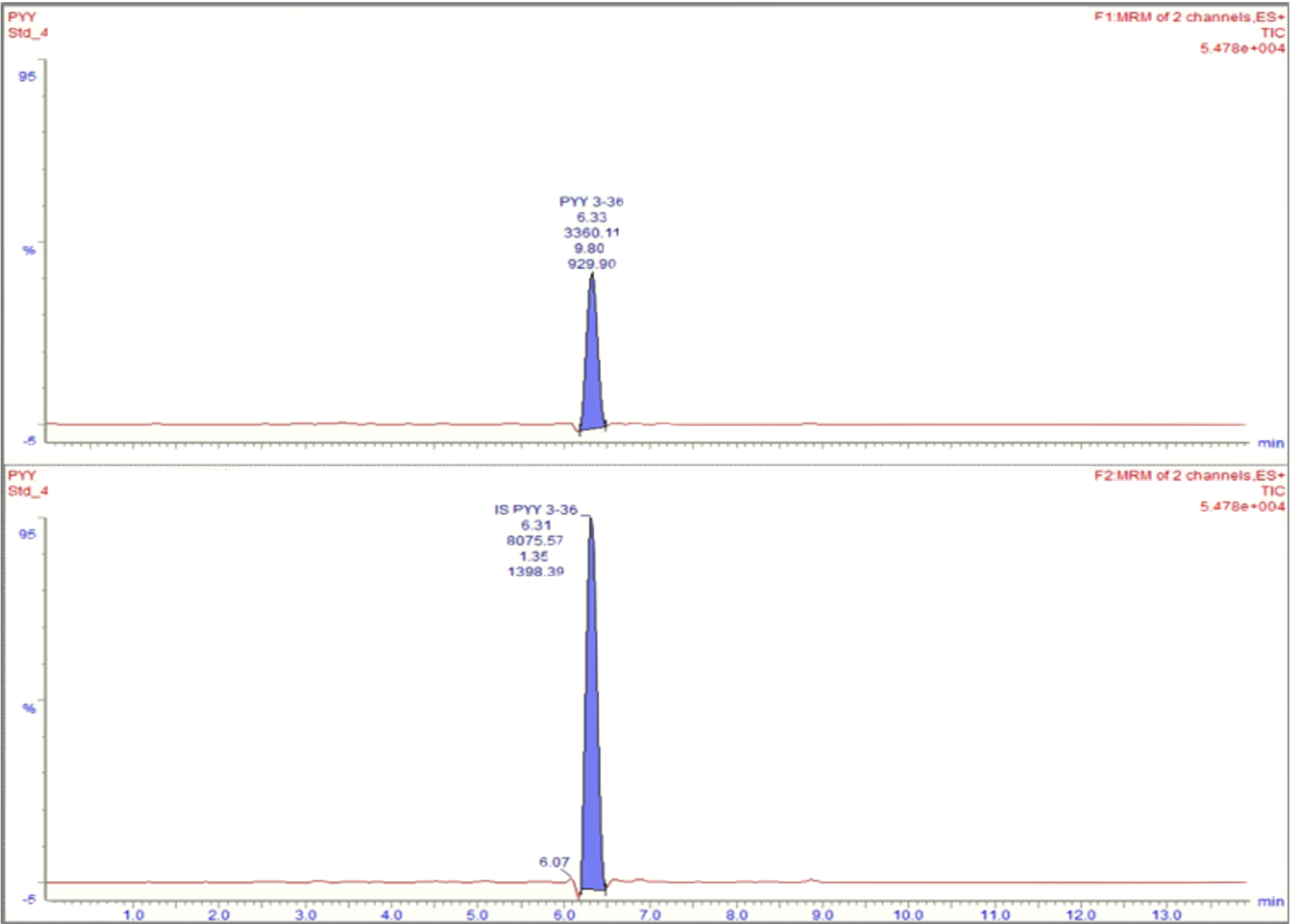  **2d**  **2D** |
| Subject sample – pre-operation  during the MMT collected at 15 mins | 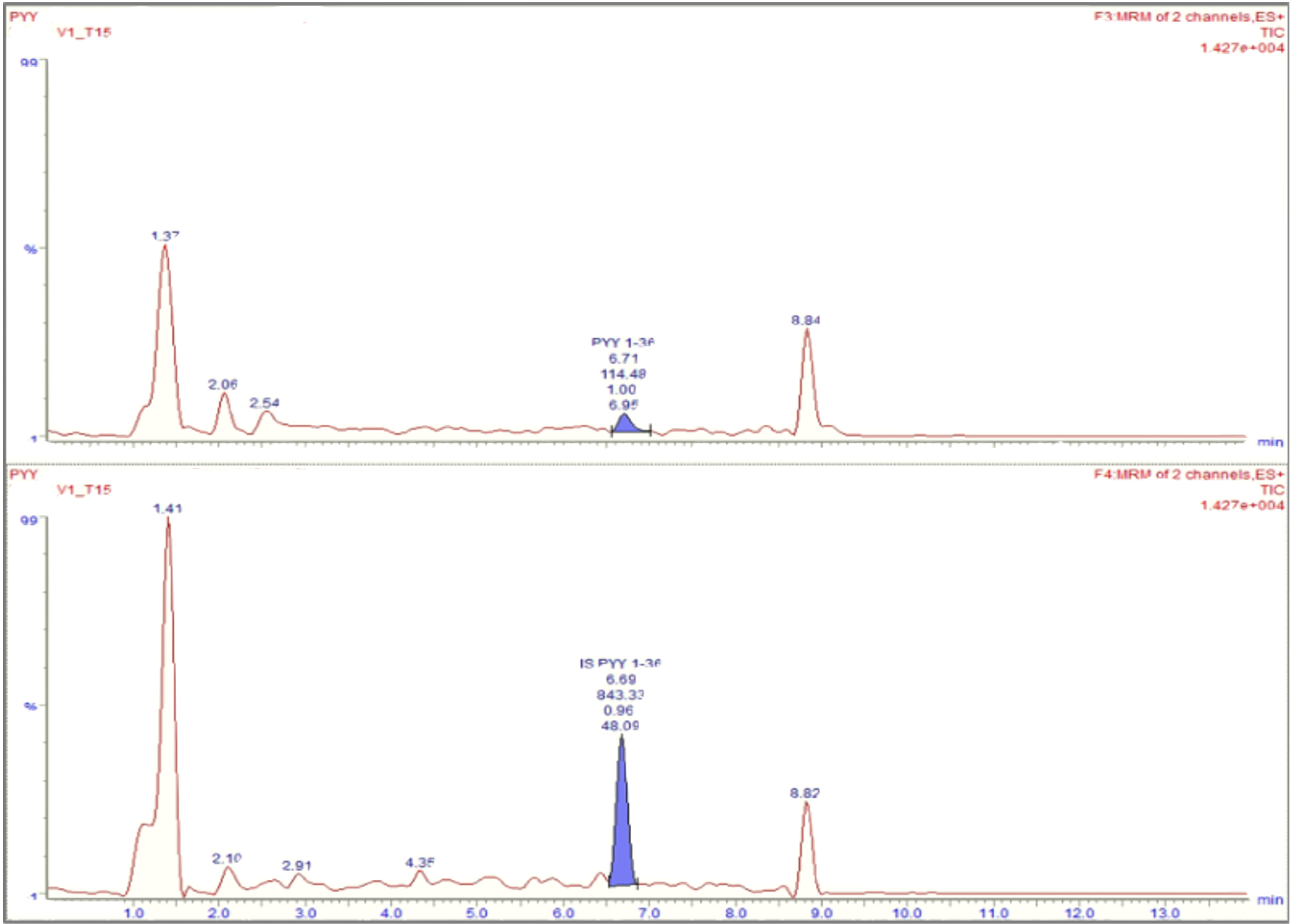  **1e**  **1E** | 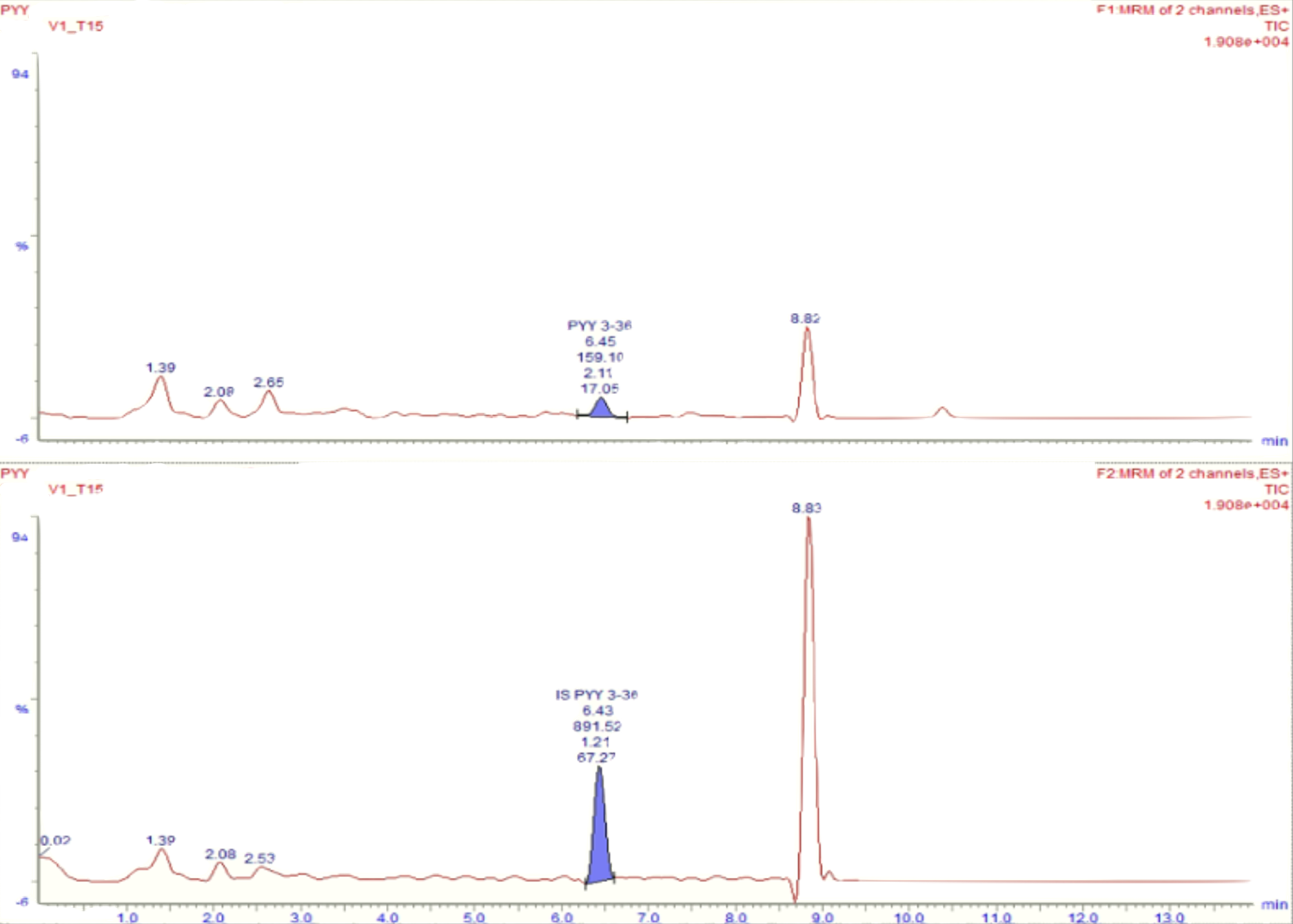  **2e**  **2E** |
| Subject sample – 1 year post-operation  during the MMT collected at 15 mins | 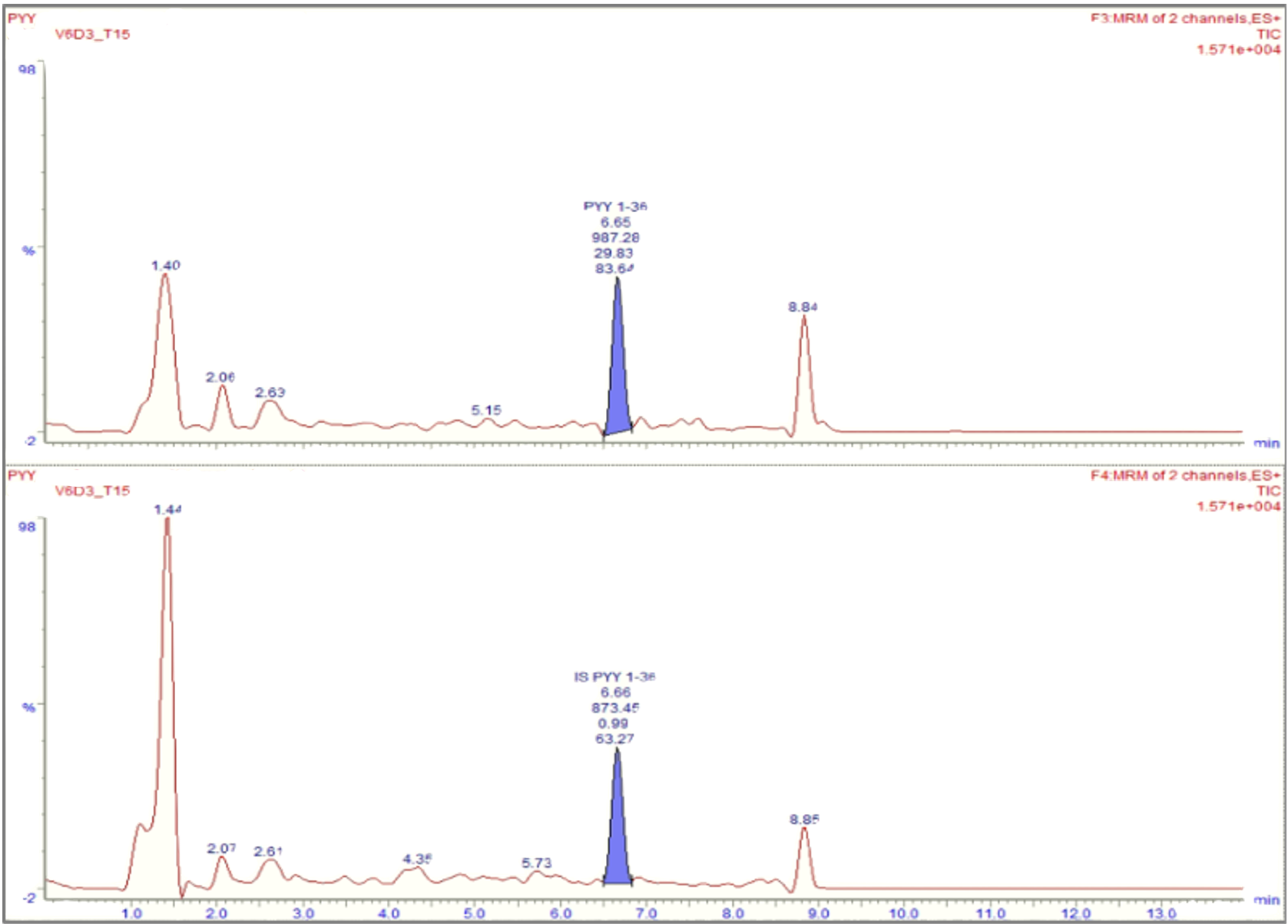  **1f**  **1F** | 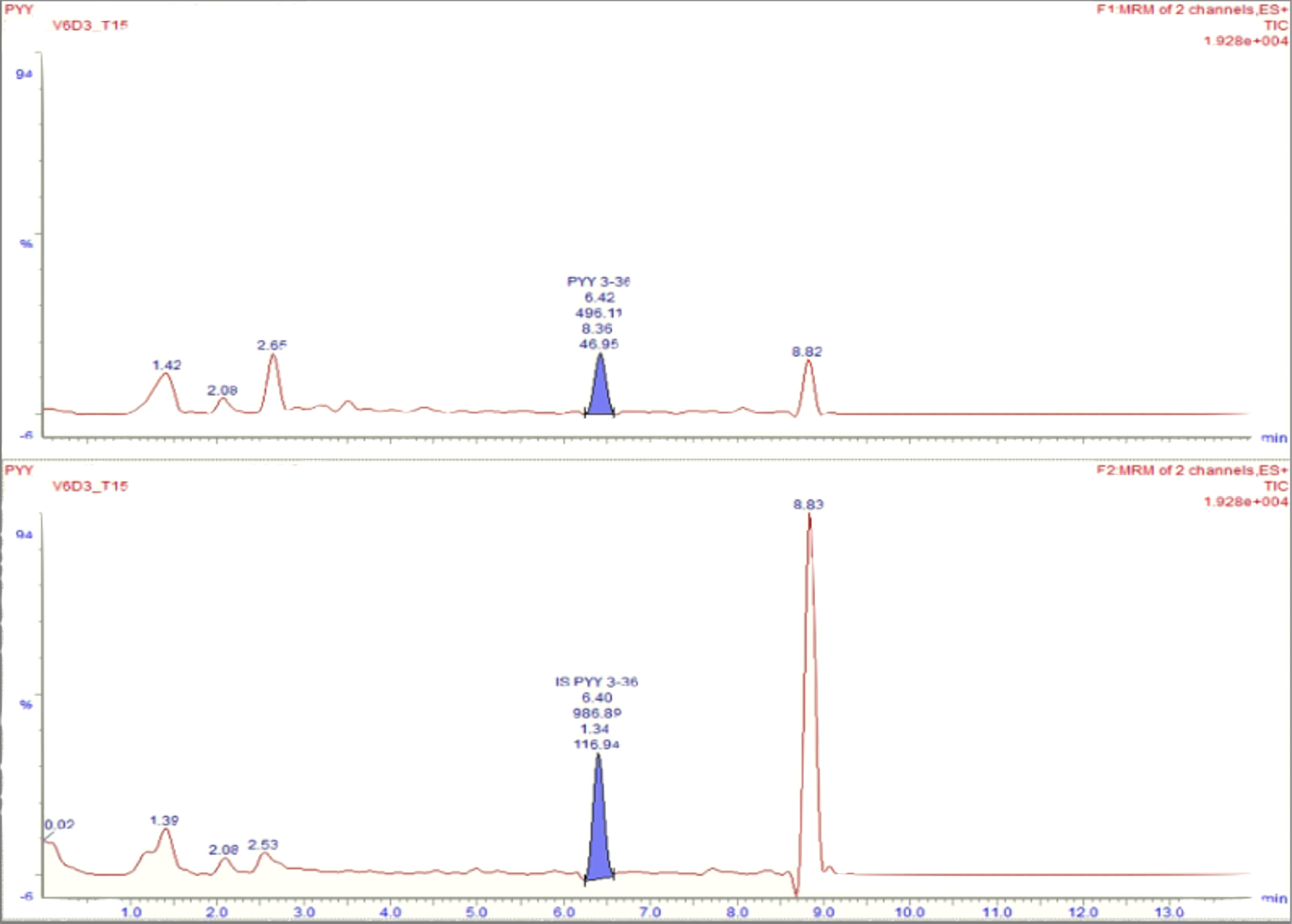  **2f**  **2F** |
| Figure 1. Total Ion Count (TIC) chromatogram examples: 1A –chromatogram of PYY_1-36_ peak in extracted Blank (plasma ‘zero’) and corresponding IS peak – PYY_1-36_ IS (1a);  2A –chromatogram of PYY_3-36_ peak in extracted Blank (plasma ‘zero’) and corresponding IS peak – PYY_3-36_ IS (2a);  1B –chromatogram of PYY_1-36_ peak in extracted plasma-based calibration standard at the level of LLOQ (2pM) and corresponding IS peak – PYY_1-36_ IS (1b);  2B –chromatogram of PYY_3-36_ peak in extracted plasma-based calibration standard at the level of LLOQ (2pM) and corresponding IS peak – PYY_3-36_ IS (2b);  1C –chromatogram of PYY_1-36_ peak in extracted plasma-based calibration standard at the level of 10pM and corresponding IS peak – PYY_1-36_ IS (1c);  2C –chromatogram of PYY_3-36_ peak in extracted plasma-based calibration standard at the level of 10pM and corresponding IS peak – PYY_3-36_ IS (2c);  1D –chromatogram of PYY_1-36_ peak in extracted solvent(20BMA)-based calibration standard at the level of 10pM and corresponding IS peak – PYY_1-36_ IS (1d);  2D –chromatogram of PYY_3-36_ peak in extracted solvent(20BMA)-based calibration standard at the level of 10pM and corresponding IS peak – PYY_3-36_ IS (2d);  1E –chromatogram of PYY_1-36_ peak in extracted subject’s plasma collected pre-op during the MMT, TP = 15 mins and corresponding IS peak – PYY_1-36_ IS (1e);  2E –chromatogram of PYY_3-36_ peak in extracted subject’s plasma collected pre-op during the MMT, TP = 15 mins and corresponding IS peak – PYY_3-36_ IS (2e);  1F –chromatogram of PYY_1-36_ peak in extracted subject’s plasma collected post-op during the MMT, TP = 15 mins and corresponding IS peak – PYY_1-36_ IS (1f);  2F –chromatogram of PYY_3-36_ peak in extracted subject’s plasma collected post-op during the MMT, TP = 15 mins and corresponding IS peak – PYY_3-36_ IS (2f);  Abbreviations: IS- Internal Standard; pM- pmol/L; 20BMA -20 µg/mL bovine serum albumin (BSA) prepared in a 1:2:7 ratio of methanol, acetic acid and water; pre-op – pre-operation; post-op – post operation; MMT-Mixed Meal Test; TP- time-point. | | |

***Supplemental Table 1 (Precision Data)***

**Inter-assay precision** was determined by measuring replicates (n=20) for each of 3-level QCs (low, L; medium, M; and high, H) on 11 separate occasions (over 3-months).

**Intra-assay precision** was obtained from analysis of 11* replicates for each of 3-level QCs in a single analytical run.  (QCM for PYY_1-36_ had 10 replicates measured as an outlier was deleted due to technical issue)

| Precision | PYY_1-36_ | | | PYY_3-36_ | | | Mean CV% |
| --- | --- | --- | --- | --- | --- | --- | --- |
|  | QCL | QCM | QCH | QCL | QCM | QCH |  |
| Inter-assay precision CV% (n=60 per each compound) | 12.01 | 11.51 | 11.55 | 15.09 | 13.46 | 12.46 | 12.68 |
| Intra-assay precision CV% (PYY1-36, n=32; PYY3-36, n=33) | 14.60 | 13.46* | 10.81 | 10.24 | 14.80 | 8.43 | 12.06 |
| Supplemental Table 1. Precision data: intra- and inter-assay precision coefficient of variation %. * 1 replicate of QCM for PYY_1-36_ was excluded from the calculations due to technical issue. | | | | | | | |

***Supplemental Figure 2 (Linearity)***

**Linear range of calibration curve** was determined by running a concertation range of calibration standards including blank plasma sample 0, and 2, 5, 10, 25, 50 and 100 pmol/L. Where subject’s results were observed above the measuring range of the assay (100 pmol/L), repeat analysis of sample diluted with 20BMA was performed. The method is linear up to 100 pmol/L for each compound (Supplemental Figure 2a and Figure 2b).

**Dilutional linearity** of the assay was determined using plasma sample spiked with 100 pmol/L of each compound, which was then serially diluted with 20BMA to 75, 50, 25 and 10 pmol/L. Each point was run in triplicates. Dilution series demonstrated good linearity and suitability of 20BMA as a diluent for the plasma samples with concentrations of PYY above 100 pmol/L. Supplemental Figure 2 demonstrates acceptable dilutional linearity up to 100 pmol/L for PYY_1-36_ (c) and PYY_3-36_ (d).


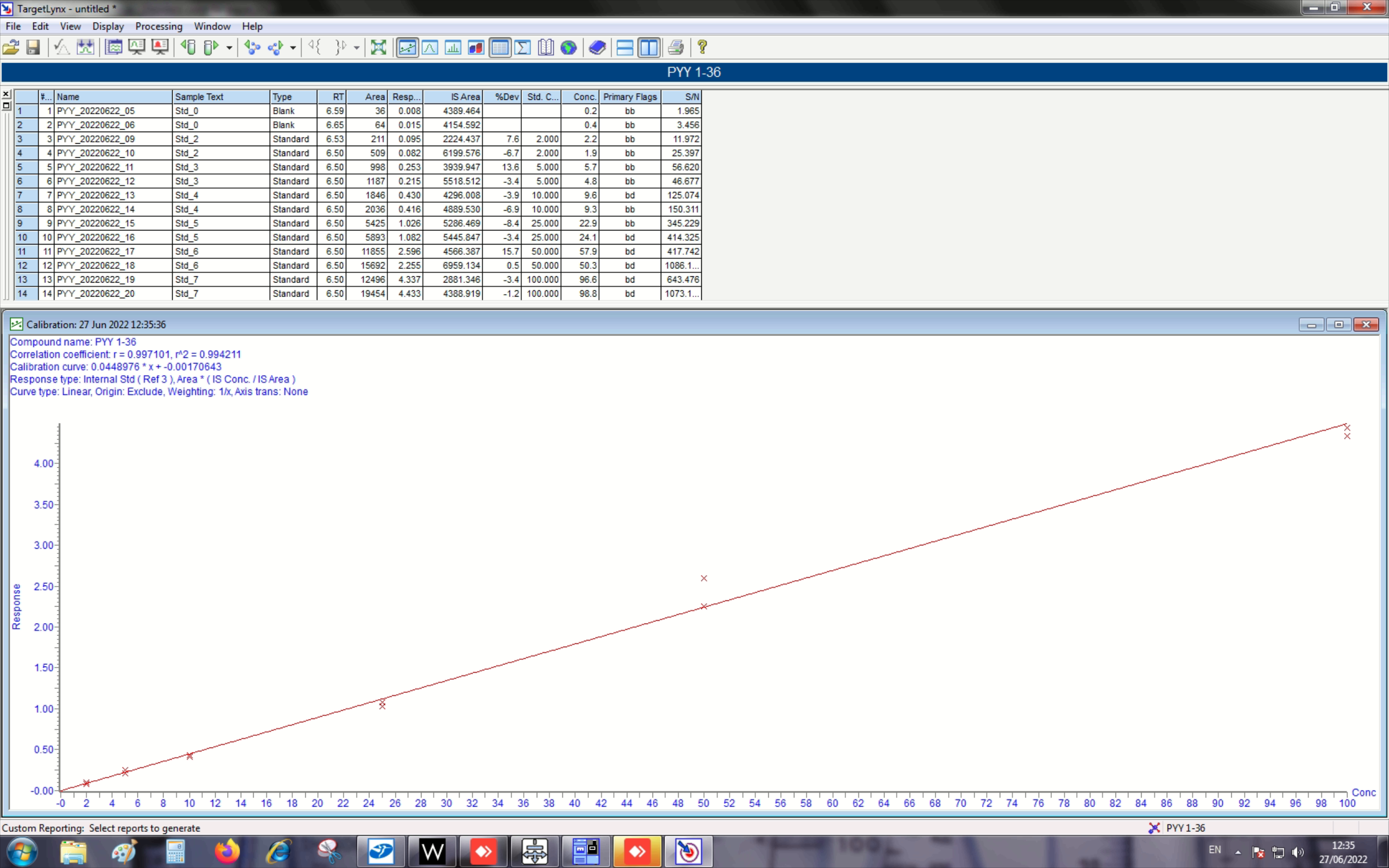


Figure 2a


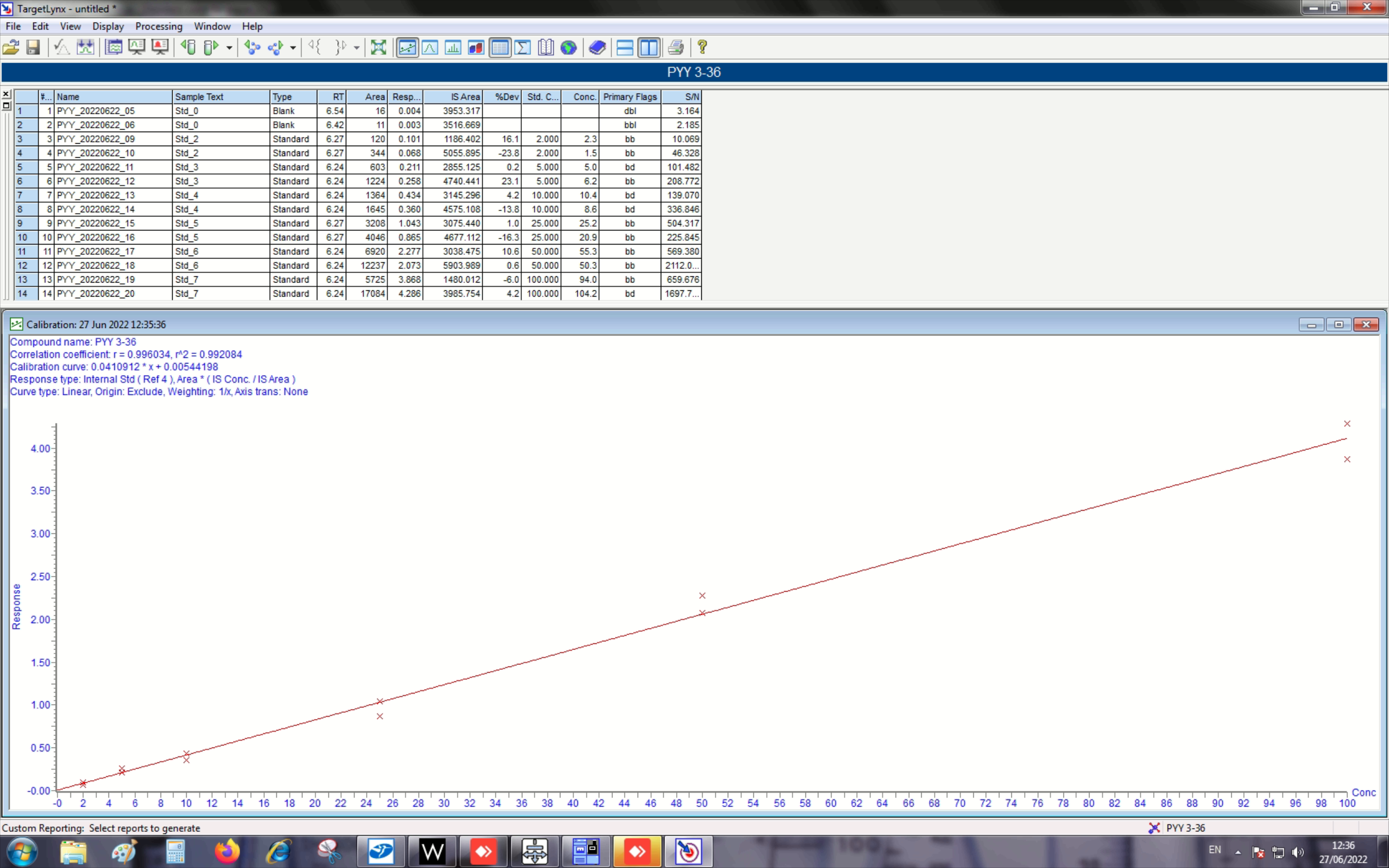


Figure 2b

Figure 2c

Figure 2d

Figure 2. PYY_1-36_ (a) and PYY_3-36_ (b) calibration curve, where X-axis represents nominal concentration and Y-axis represents responses. Linear range of dilutions for PYY_1-36_ (c) and PYY_3-36_ (d), where X-axis represents nominal concentration and Y-axis calculated concentrations.

***Supplemental Table 2 and 3 (Matrix Effects and Extraction Recovery)***

**Extraction recovery** for plasma samples and between different matrices was assessed (Supplemental Table 2). **Matrix effect (ME)** experiments showed that ion suppression was adequately compensated for by internal standards (Supplemental Table 3).

| Extraction recovery in plasma | PYY_1-36_ | | PYY_3-36_ | | Extraction recovery in 20BMA and plasma | PYY_1-36_ | | PYY_3-36_ | |
| --- | --- | --- | --- | --- | --- | --- | --- | --- | --- |
| Concentration (pM) | % Recovery | Mean % Recovery | % Recovery | Mean % Recovery | Concentration (pM) | % Recovery | Mean % Recovery | % Recovery | Mean % Recovery |
| 0 | 119.04 | 85.167 | 92.86 | 92.13 | 0 | 117.12 | 108.22 | 89.19 | 85.44 |
| 2 | 126.61 |  | 107.94 |  | 2 | 115.92 |  | 66.79 |  |
| 5 | 59.26 |  | 108.42 |  | 5 | 73.03 |  | 75.12 |  |
| 50 | 78.58 |  | 78.13 |  | 50 | 128.23 |  | 92.89 |  |
| 100 | 76.22 |  | 74.03 |  | 100 | 106.80 |  | 103.23 |  |
| Supplemental Table 2:  **Extraction recovery** for plasma samples utilising the average peak areas ^1^ and between different matrices using mean responses ^2,3^. | | | | | | | | | |

| Matrix effects | PYY_1-36_ | | PYY_3-36_ | |
| --- | --- | --- | --- | --- |
| Concentration (pM) | % ME | Mean %ME | % ME | Mean %ME |
| 5 | 97.56 | 99.61 | 77.66 | 91.17 |
| 50 | 99.14 |  | 97.66 |  |
| 100 | 102.14 |  | 98.20 |  |
| Supplemental Table 3: **Matrix effect (ME)** was measured at three concentrations (5, 50, 100 pmol/L) for each of PYY species | | | | |

***Supplemental Table 4 (Limits of Quantification)***

The **Low Limits of Quantification (LLOQ)** for PYY_1-36_ and PYY_3-36_ was determined by analyses of 10 replicates of spiked blank plasma samples with 2 pmol/L, 5 pmol/L and 10 pmol/L. Derived LLOQ for PYY_1-36_ and PYY3-36 was the lowest concentration that could be measured with <20% precision, signal:noise ratio >1:10 and an instrument response five times greater than the blank, 2.4 pmol/L and 2.0 pmol/L respectively. Supplemental Table 4 shows the details of LLOQ.

| **LLOQ** | PYY_1-36_ | | | | PYY_3-36_ | | | |
| --- | --- | --- | --- | --- | --- | --- | --- | --- |
| Concentration [pM] | Mean concentration | SD | CV% | S/N | Mean concentration | SD | CV% | S/N |
| 1.00 | 0.97 | 0.47 | 48.02 | 4.28 | 1.16 | 0.46 | 39.81 | 15.88 |
| 2.00 | 2.44 | 0.46 | 18.85 | 12.90 | 2.04 | 0.36 | 17.63 | 29.25 |
| 5.00 | 4.95 | 0.74 | 14.80 | 22.34 | 5.19 | 0.90 | 17.32 | 39.99 |
| Supplemental Table 4: The Low Limits of Quantification (LLOQ) for PYY_1-36_ and PYY_3-36_. | | | | | | | | |

***Supplemental Figure 3 (Carry-over Experiment)***

Experiments were carried out to detect any carry-over by measuring five 1 pmol/L spikes (below LLOQ) followed by 10 injections alternating between 100 pmol/L spike (the highest concentration of calibration standard) and 1 pmol/L with the results for 1 pmol/L expected to be within 10% of the initial five injections of the low concentration sample and ≥25% of LLOQ. Negligible carryover was observed for each analyte (Supplemental Figure 3).

***
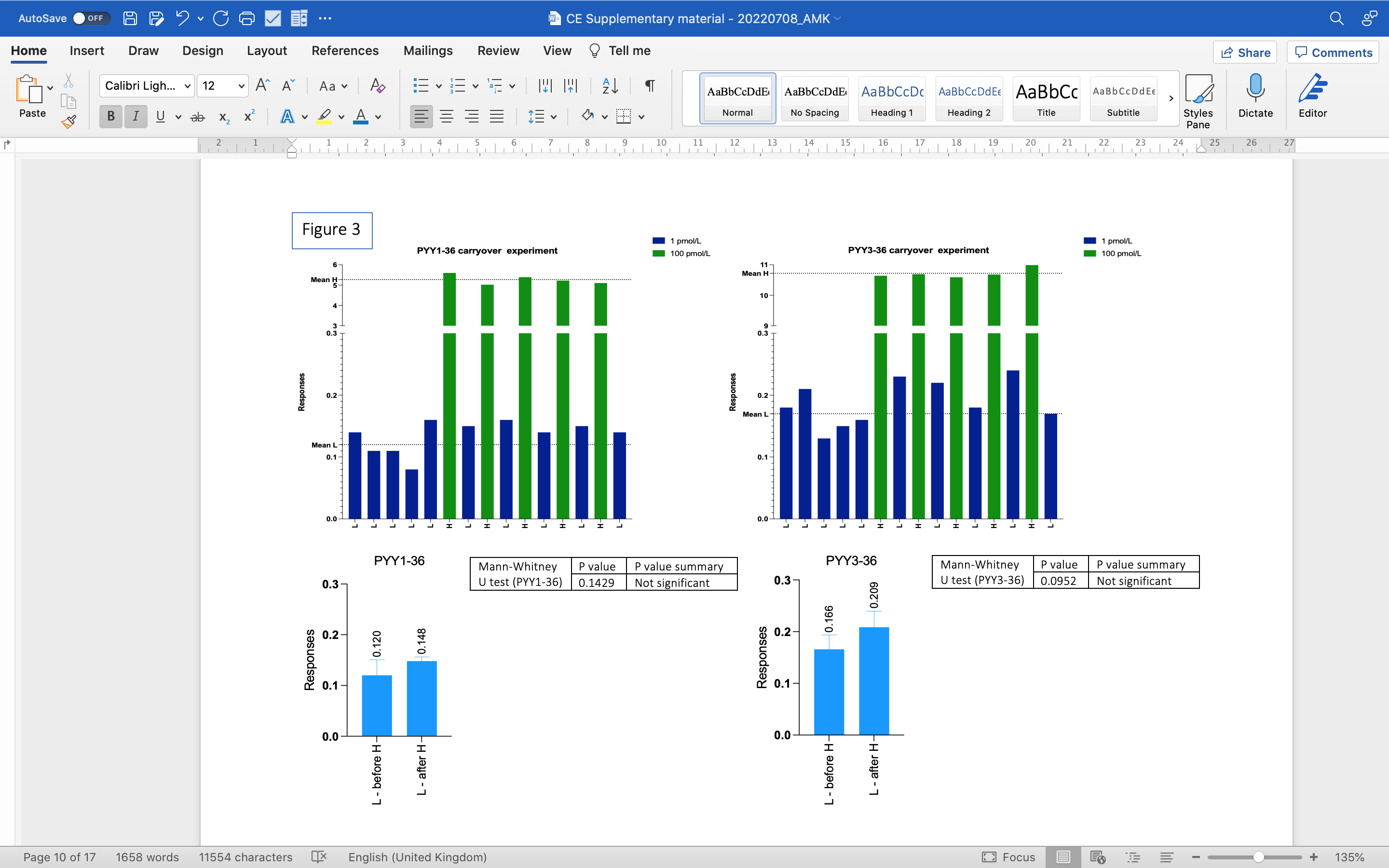
***

Supplemental Figure 3. PYY carryover experiments; non-significant carryover detected for each compound.

***Supplemental Figure 4 (Specificity and Interferences)***

PYY_1-36_ and PYY_3-36_ were also measured in the presence of PYY_1-34_, PYY_3-34_, PP and NPY to confirm the specificity of the assay. This experiment was carried out to show the ability of the mass spectrometer to distinguish between these very closely related compounds, where the difference in molecular weight between PYY_1-36_ and its internal standard can be only 28 Da. No changes to the analyte target values were detected.

|  | Extracted spiked plasma ‘zero’ sample with PYY_1-36_, concentration 50pmol/L | Extracted spiked plasma ‘zero’ sample with PYY_3-36_, concentration 50pmol/L |
| --- | --- | --- |
| 50pmol/L of the peptide | 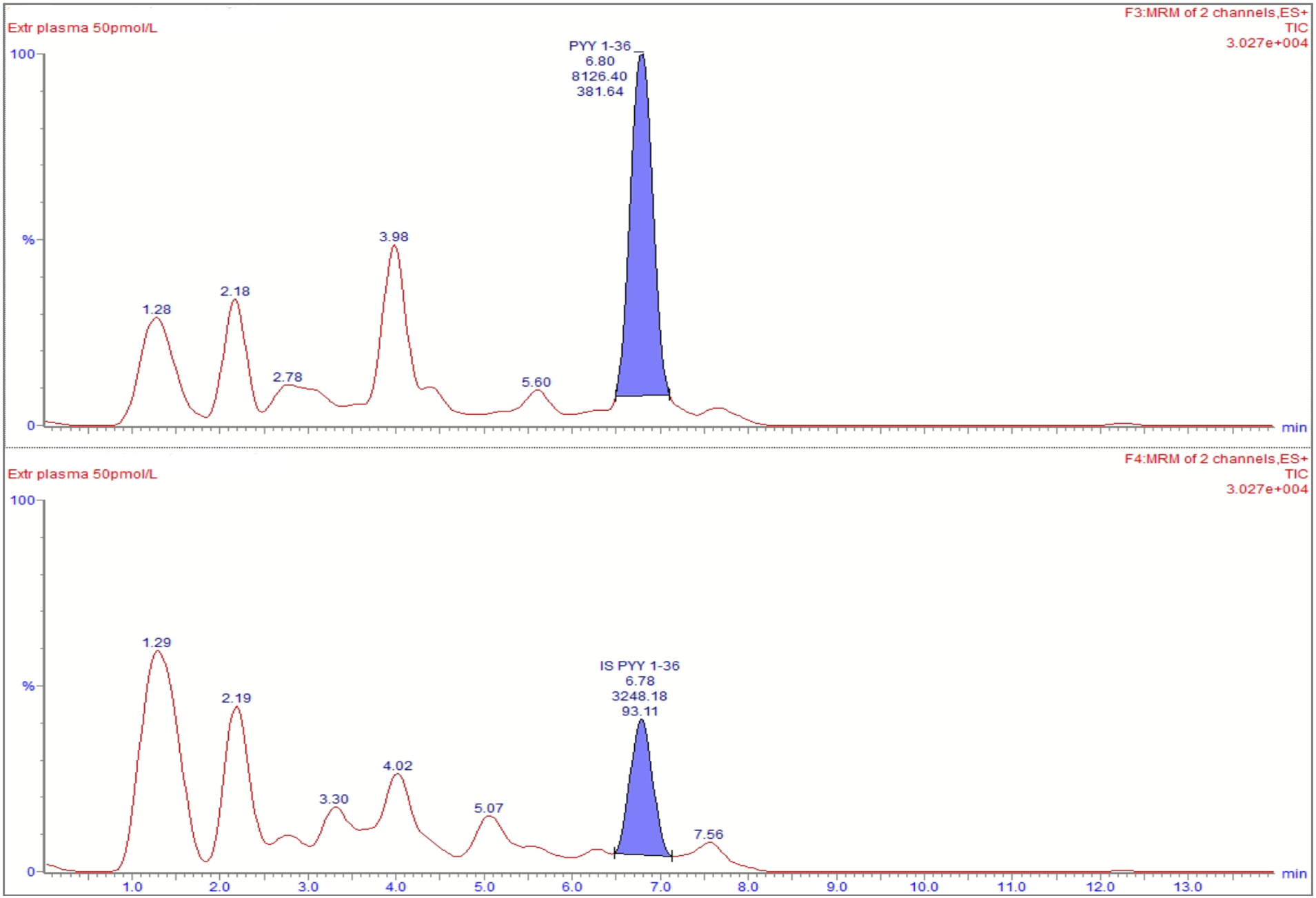 | 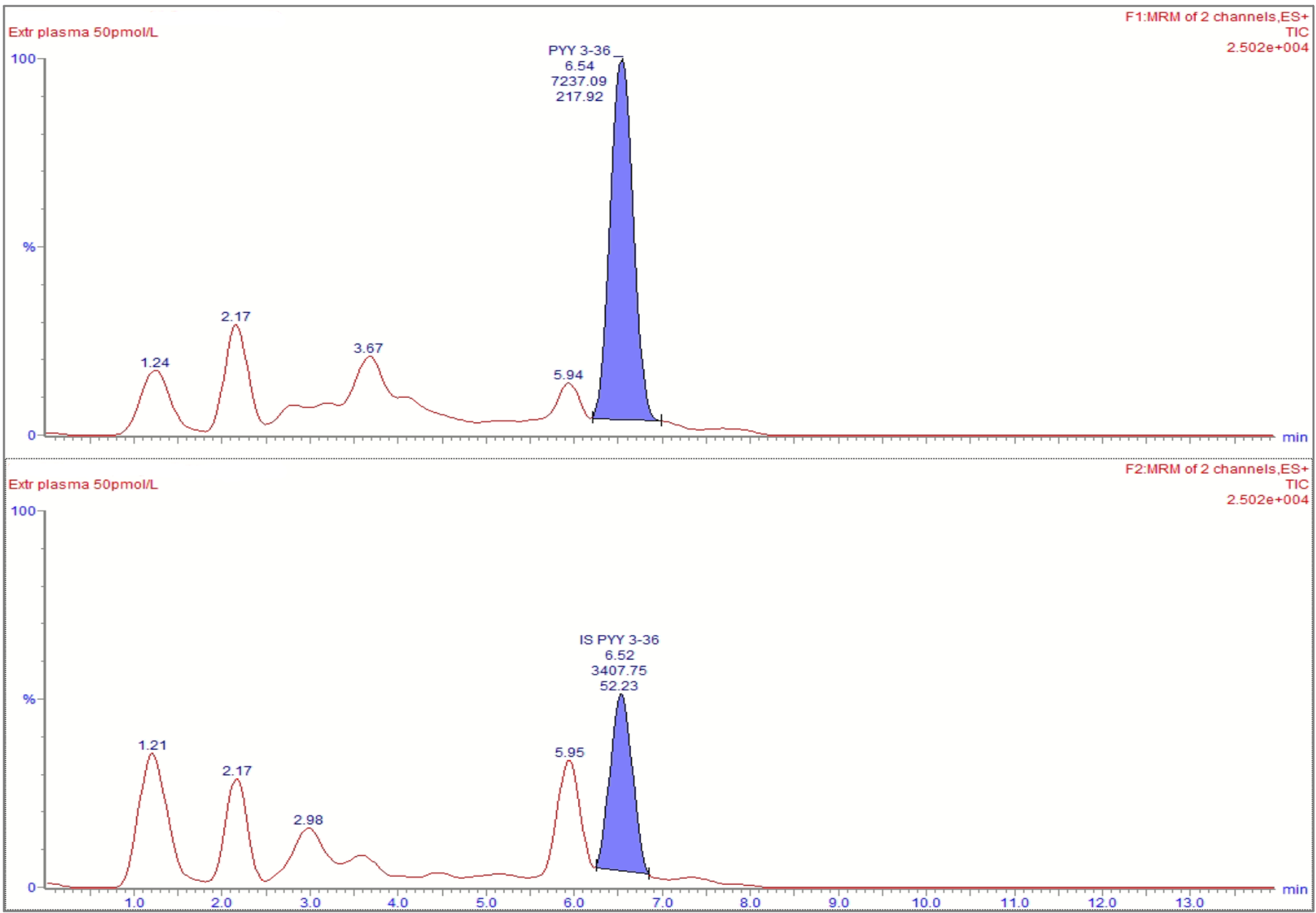 |
| + 50pmol/L of PYY_1-34_ | 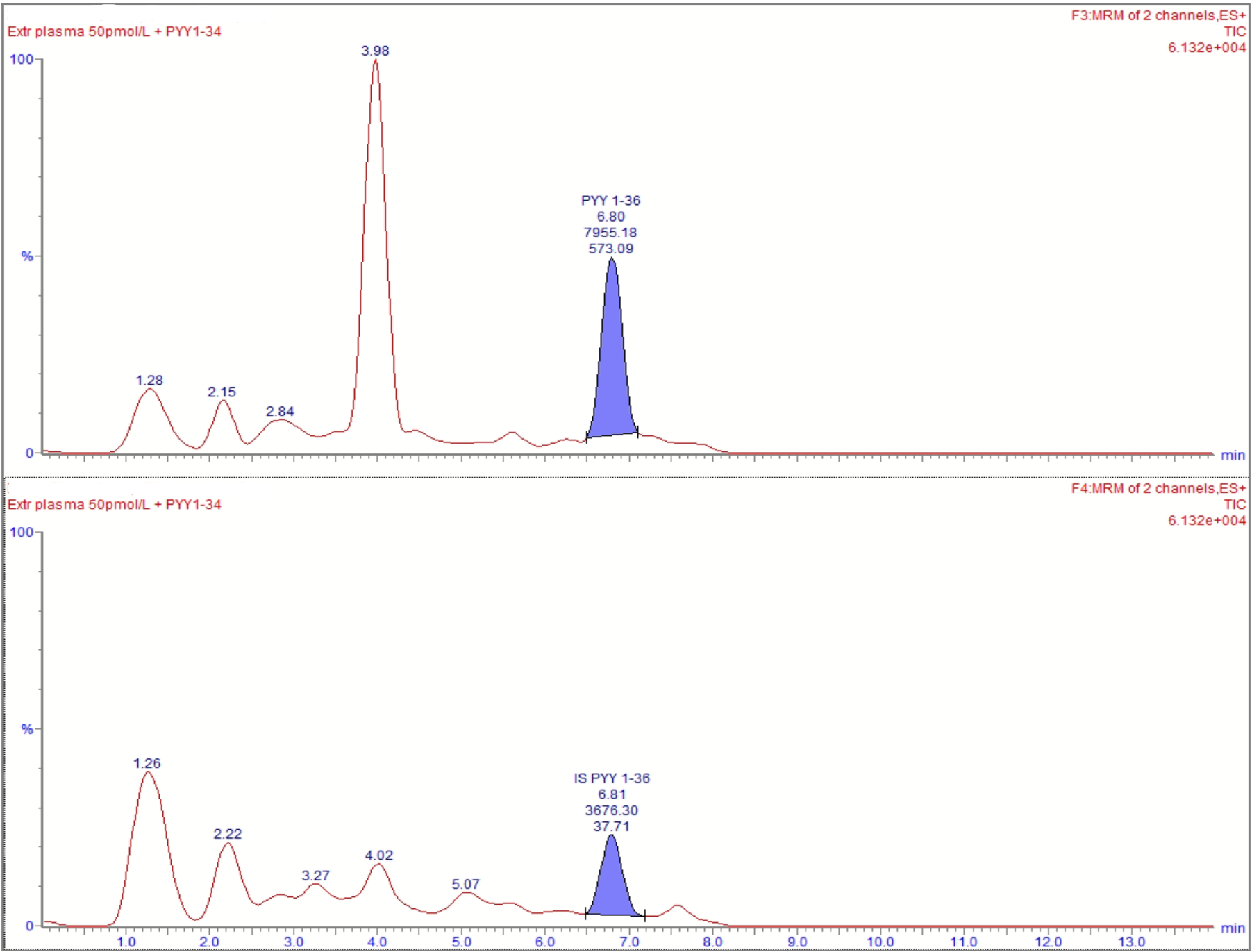 | 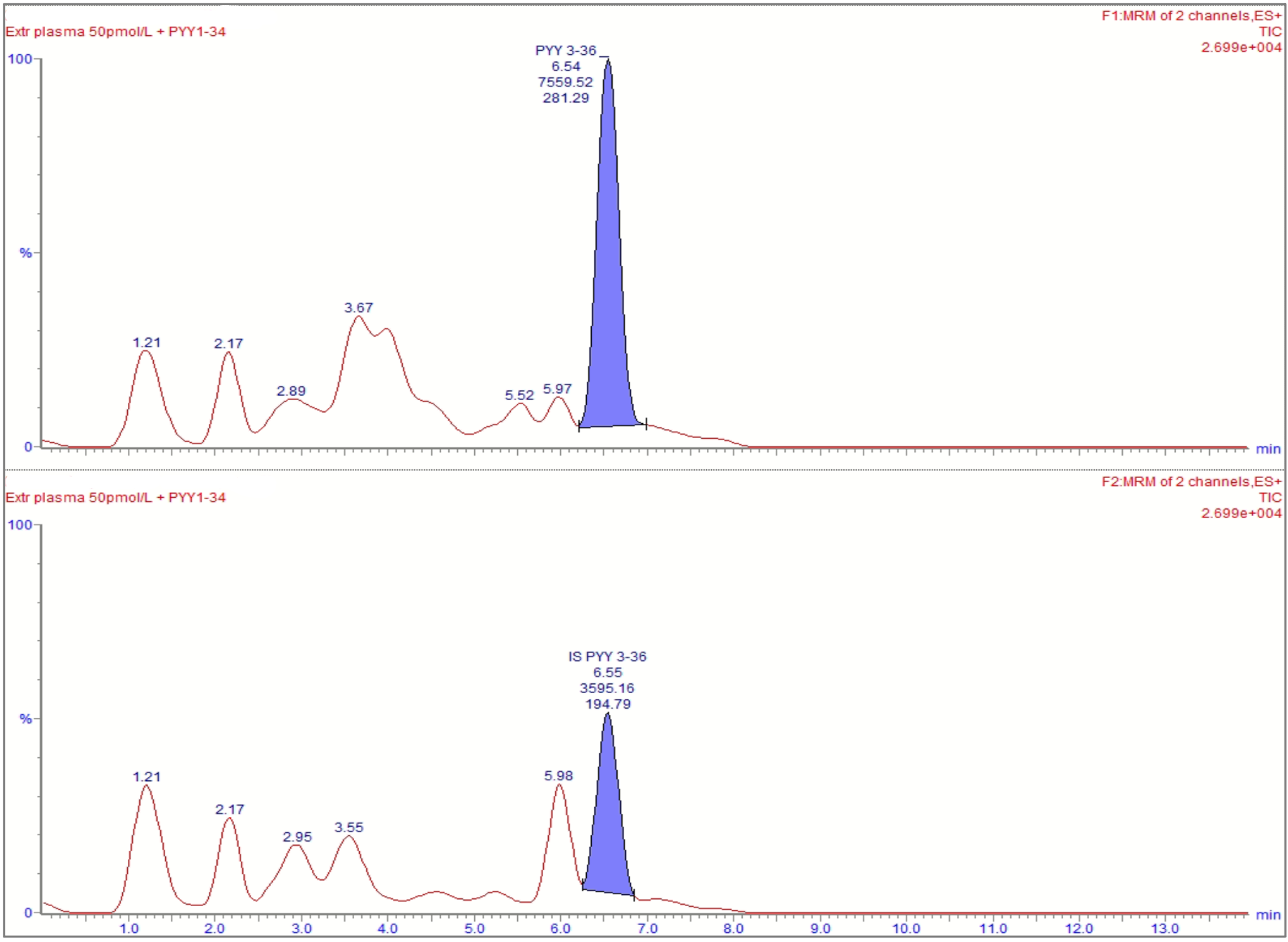 |
| + 50pmol/L of PYY_3-34_ | 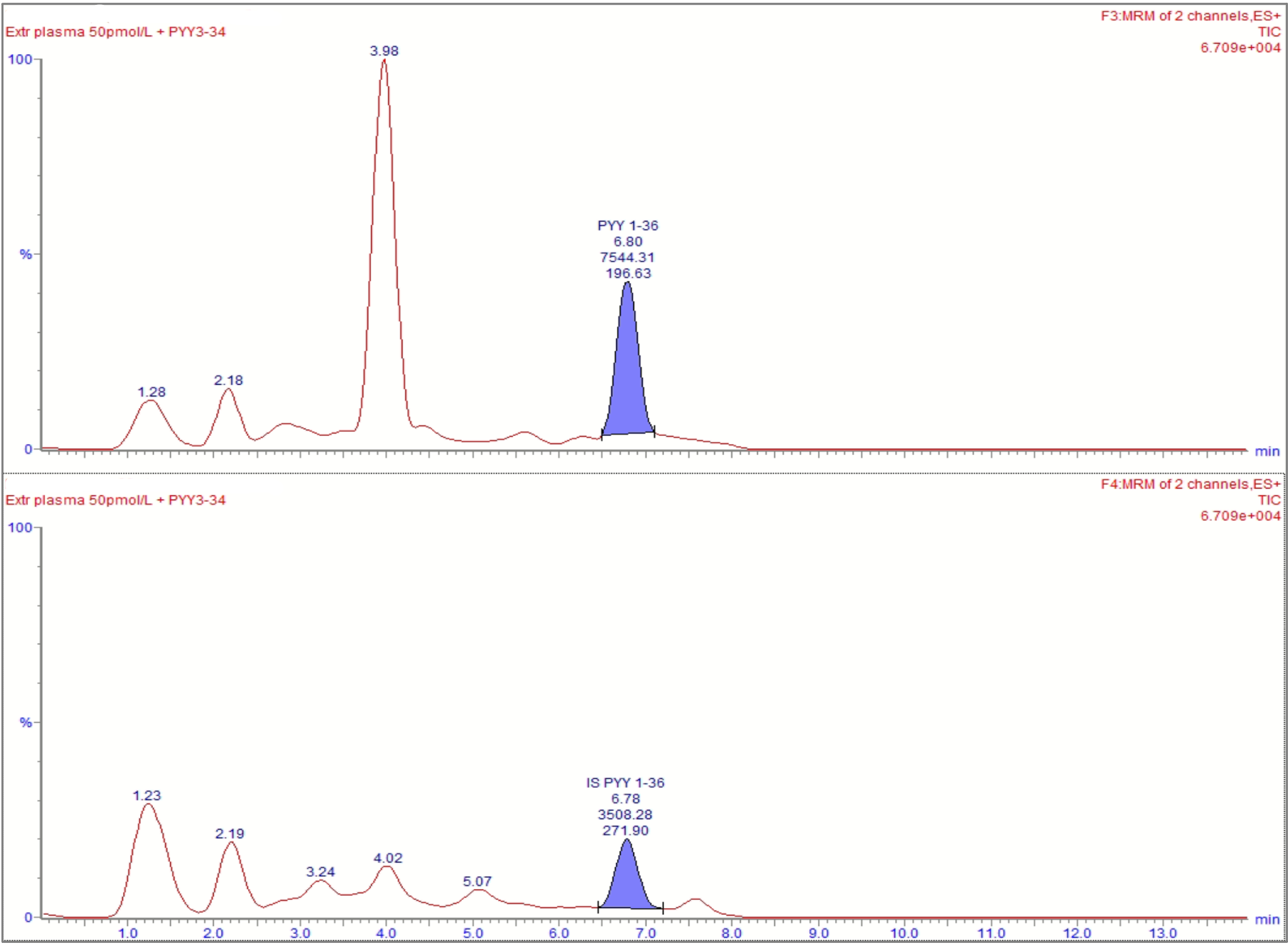 | 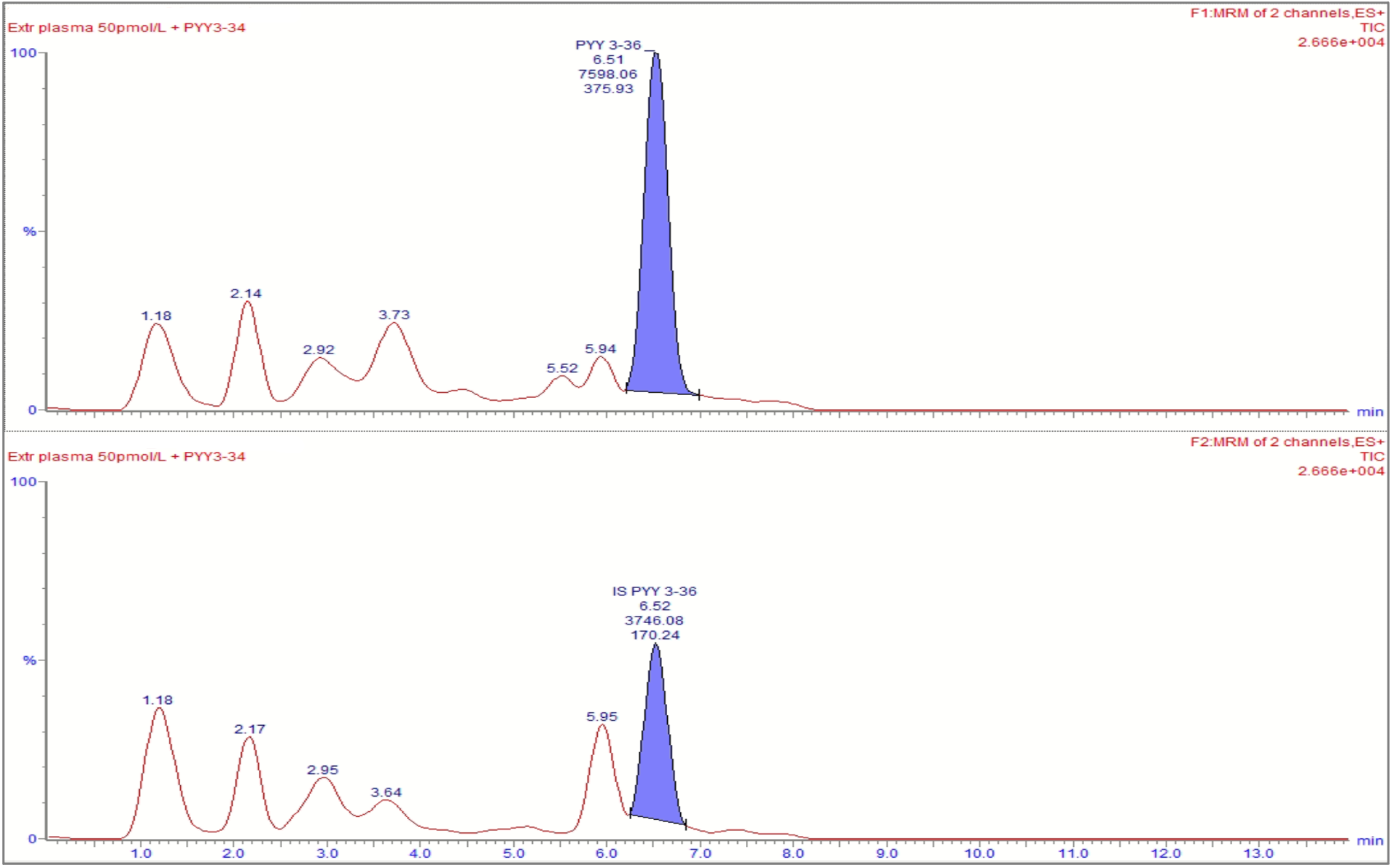 |
| + 50pmol/L of PP | 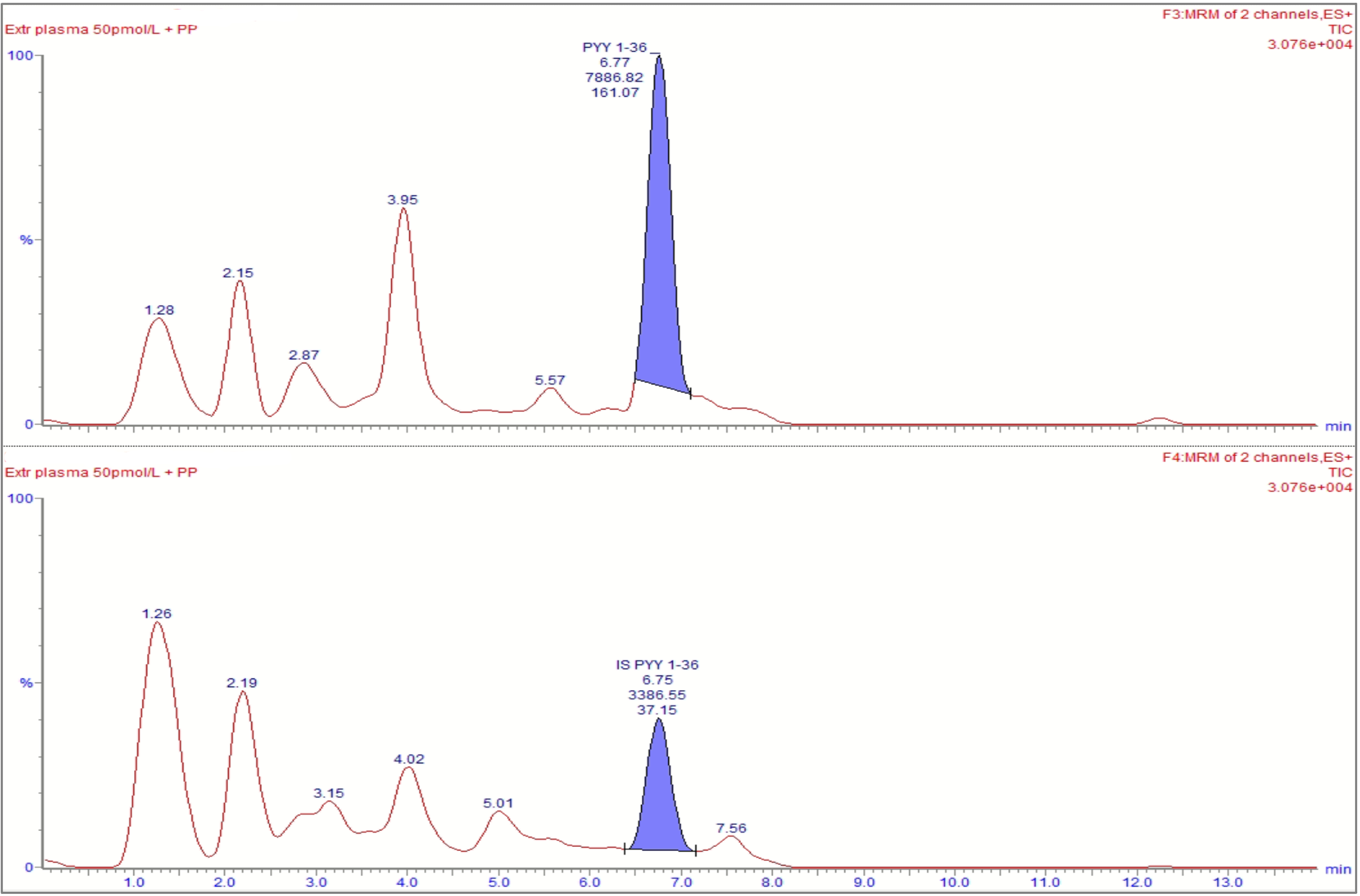 | 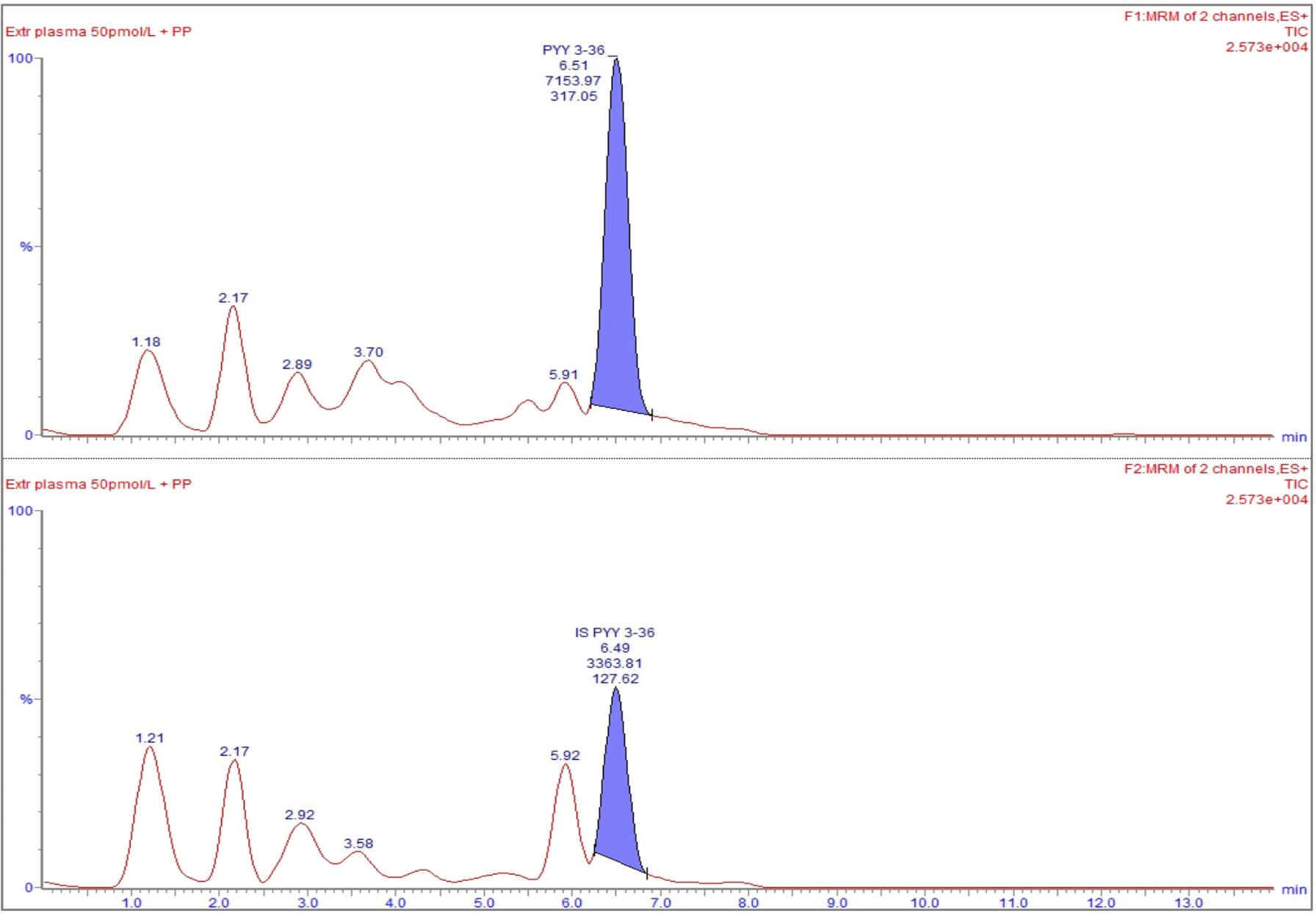 |
| + 50pmol/L of NPY | 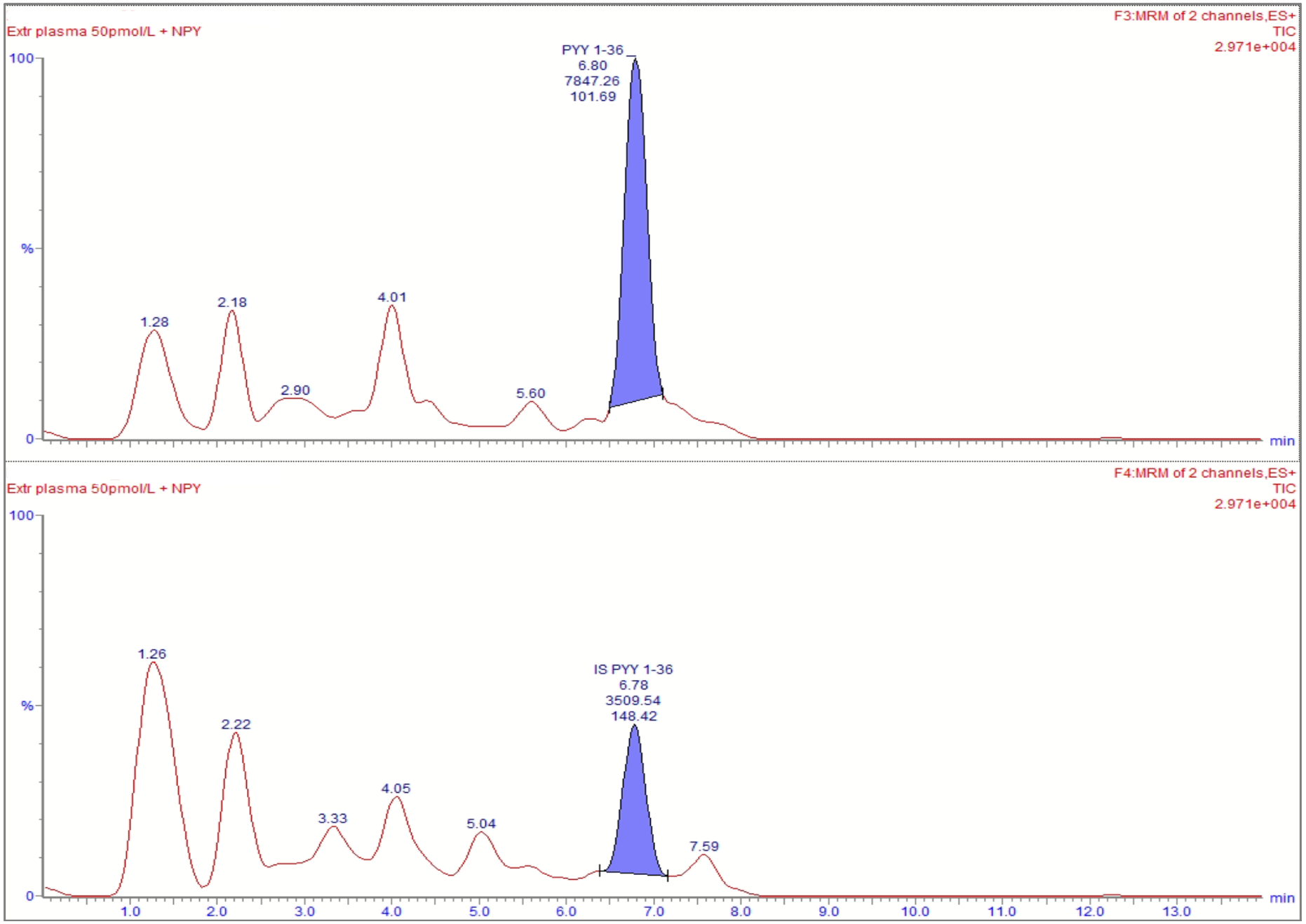 | 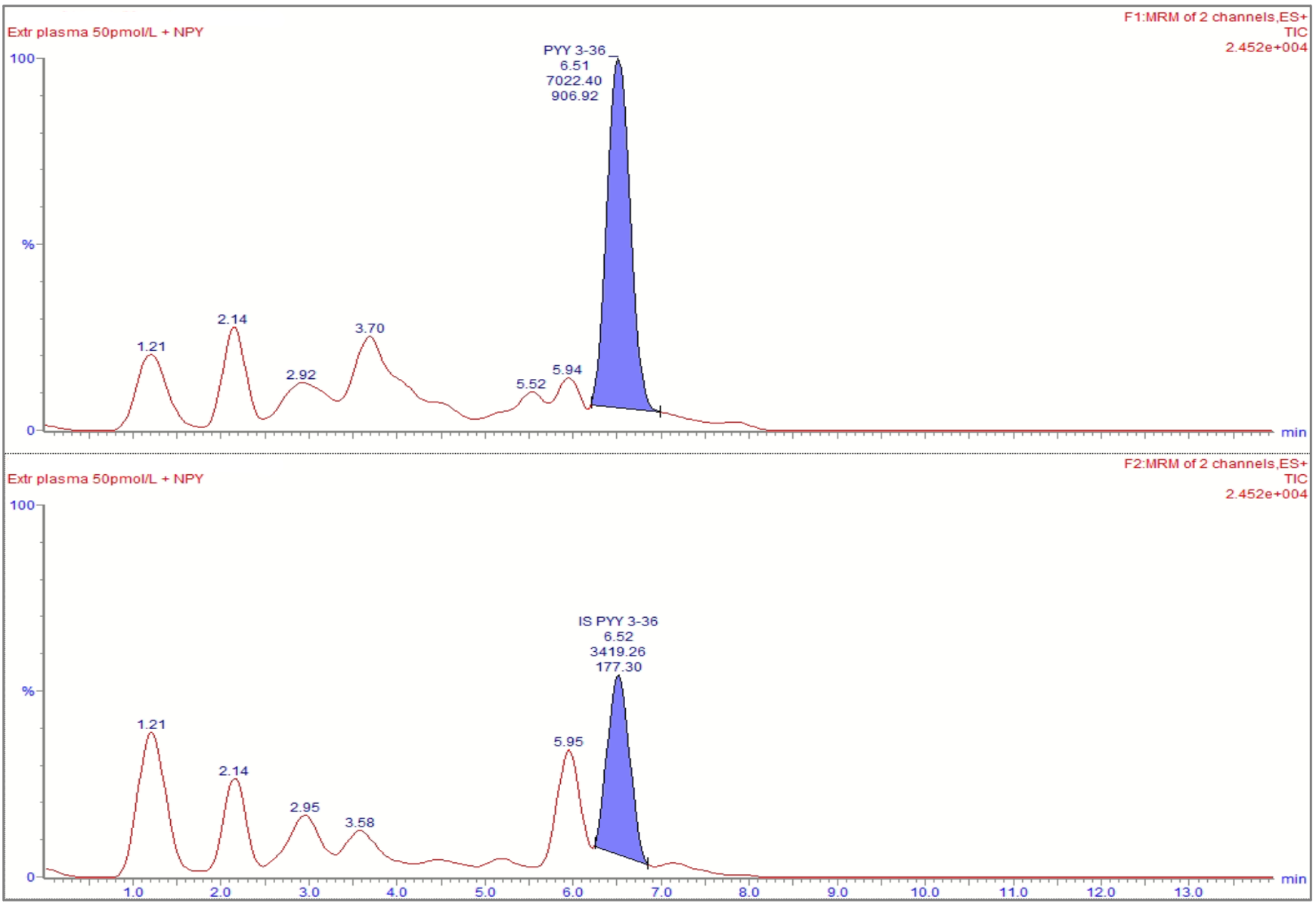 |
| Supplemental Figure 4. Specificity of LC-MS/MS assay to PYY_1-36_ and PYY _3-36_. PYY_1-36_ and PYY_3-36_ and corresponding Internal Standard peaks are in blue. No significant changes to target values of both compounds (PYY_1-36_ and PYY_3-36_) were detected when tested in presence of 50 pmol/L of potential interferant. NPY – neuropeptide Y; PP – Pancreatic Polypeptide. | | |

^3–7^

***References***

1. Nouman EG, Al-Ghobashy MA, Lotfy HM. Development and validation of LC-MS/MS assay for the determination of Butoconazole in human plasma: Evaluation of systemic absorption following topical application in healthy volunteers. *Bull Fac Pharmacy, Cairo Univ*. 2017;55(2):303-310. doi:10.1016/j.bfopcu.2017.04.003

2. Alegete P, Kancherla P, Albaseer SS, Boodida S. A fast and reliable LC-MS/MS method for simultaneous quantitation of fluoxetine and mirtazapine in human plasma. *Anal Methods*. 2014;6(18):7407-7414. doi:10.1039/c4ay01057d

3. Rower JE, Bushman LR, Hammond KP, Kadam RS, Aquilante CL. Validation of an LC/MS method for the determination of gemfibrozil in human plasma and its application to a pharmacokinetic study. *Biomed Chromatogr*. 2010;24(12):1300-1308. doi:10.1002/bmc.1440

4. Spectrometry LC, Guideline A. *C62-A*.; 2014.

5. Lynch KL. CLSI C62-A: A new standard for clinical mass spectrometry. *Clin Chem*. 2016;62(1):24-29. doi:10.1373/clinchem.2015.238626

6. Smith G. European medicines agency guideline on bioanalytical method validation: What more is there to say? *Bioanalysis*. 2012;4(8):865-868. doi:10.4155/bio.12.44

7. U.S. Department of Health and Human Services. Food and Drug Administration. Guidance for Industry: Bioanalytical Method Validation. *Fda*. 2001;(May):4-10.

8. NICE. Identification, assessment and management of overweight and obesity in children, young people and adults - Partial update of CG43 Methods, evidence and recommendations. November 2014. 2014;(November):1-154.


Supplement: Supplementary file 1 — Supporting information. [file CEN-99-272-s001.docx]
